# Phenotypic and Multi-Omics Characterization of *Escherichia coli* K-12 Adapted to Chlorhexidine Identifies the Role of MlaA and Other Cell Envelope Alterations Regulated by Stress Inducible Pathways in CHX Resistance

**DOI:** 10.3389/fmolb.2021.659058

**Published:** 2021-05-19

**Authors:** Branden S. J. Gregorchuk, Shelby L. Reimer, Kari A. C. Green, Nicola H. Cartwright, Daniel R. Beniac, Shannon L. Hiebert, Timothy F. Booth, Patrick M. Chong, Garrett R. Westmacott, George G. Zhanel, Denice C. Bay

**Affiliations:** ^1^Department of Medical Microbiology and Infectious Diseases, University of Manitoba, Winnipeg, MB, Canada; ^2^National Microbiology Laboratory, Public Health Agency of Canada, Winnipeg, MB, Canada

**Keywords:** chlorhexidine, retrograde phospholipid transport, disinfectant, *Escherichia coli*, MlaA, antiseptic resistance, multi-omics, antimicrobial resistance (AMR)

## Abstract

Chlorhexidine (CHX) is an essential medicine used as a topical antiseptic in skin and oral healthcare treatments. The widespread use of CHX has increased concerns regarding the development of antiseptic resistance in Enterobacteria and its potential impact on cross-resistance to other antimicrobials. Similar to other cationic antiseptics, resistance to CHX is believed to be driven by three membrane-based mechanisms: lipid synthesis/transport, altered porin expression, and increased efflux pump activity; however, specific gene and protein alterations associated with CHX resistance remain unclear. Here, we adapted *Escherichia coli* K-12 BW25113 to increasing concentrations of CHX to determine what phenotypic, morphological, genomic, transcriptomic, and proteomic changes occurred. We found that CHX-adapted *E. coli* isolates possessed no cross-resistance to any other antimicrobials we tested. Scanning electron microscopy imaging revealed that CHX adaptation significantly altered mean cell widths and lengths. Proteomic analyses identified changes in the abundance of porin OmpF, lipid synthesis/transporter MlaA, and efflux pump MdfA. Proteomic and transcriptomic analyses identified that CHX adaptation altered *E. coli* transcripts and proteins controlling acid resistance (*gadE, cdaR*) and antimicrobial stress-inducible pathways Mar-Sox-Rob, stringent response systems. Whole genome sequencing analyses revealed that all CHX-resistant isolates had single nucleotide variants in the retrograde lipid transporter gene *mlaA* as well as the *yghQ* gene associated with lipid A transport and synthesis. CHX resistant phenotypes were reversible only when complemented with a functional copy of the *mlaA* gene. Our results highlight the importance of retrograde phospholipid transport and stress response systems in CHX resistance and the consequences of prolonged CHX exposure.

## Introduction

Chlorhexidine (CHX) is a commonly used antiseptic and disinfectant in medical, dental, and veterinary practice and it is listed as an essential medicine by the World Health Organization (World Health Organization, 2019). CHX is the active antimicrobial ingredient used in a variety of clinical antiseptics (skin, oral, and eye washes) and daily use products (cosmetics and personal hygiene products), making CHX usage widespread. This antiseptic is a membrane-active bisbiguanide compound that consists of two cationic biguanidine moieties linked by a hexamethyl acyl-chain region (Nayyar, 2015). Similar to other hydrophobic cationic antiseptics, CHX kills and/or inhibits cell growth in a concentration dependent manner by disrupting cell membrane phospholipids by displacing divalent cations at the anionic cell membrane surface (Gilbert and Moore, 2005; Nayyar, 2015). Specifically, CHX acts by inserting itself in between phospholipid headgroup pairs gradually destabilizing cell membrane integrity by forming gaps in the phospholipid membrane bilayer that result in cell content leakage and death (Gilbert and Moore, 2005). Although bacterial resistance to CHX has not been convincingly shown at its working concentrations, Gram-negative bacterial resistance to CHX is becoming a growing concern. There are increasing reports of CHX-resistant Enterobacterales (Kampf, 2016, 2018; Cieplik et al., 2019), where CHX-resistant pathogens, *Klebsiella* spp. and *Salmonella* spp., have demonstrated cross-resistance to antibiotics, most notably the last-line polymyxin antibiotic colistin (Wand et al., 2017; Hashemi et al., 2019; Verspecht et al., 2019). Increasing resistance as well as possible antibiotic cross-resistance is very concerning given CHX’s clinical importance as a medical antiseptic and, in some cases, as a last-line debridement treatment (Brookes et al., 2020). This highlights an important knowledge gap to address regarding how intrinsic CHX mechanisms of resistance develop, especially among antimicrobial resistant species deemed to be critical priority pathogens. Unfortunately, there are no clinically defined CHX breakpoint concentrations to distinguish resistant from susceptible CHX concentrations according to the Clinical Laboratory Standards Institute (CLSI^1^) or European Committee on Antimicrobial Susceptibility Testing (EUCAST^2^). Despite the absence of defined CHX breakpoints, we will refer to reduced CHX susceptibility as “resistant” rather than “tolerant” herein based on minimum inhibitory concentration values defined for antiseptics and antibiotics (Cerf et al., 2010; Brauner et al., 2016).

Previous studies have attempted to identify CHX-resistance’s mechanisms of action using clinical isolates (Condell et al., 2012; Naparstek et al., 2012; Fernández-Cuenca et al., 2015; Vali et al., 2015) or laboratory adapted bacteria/isolates that were gradually exposed to increasing concentrations of CHX over numerous sub-cultures (Braoudaki and Hilton, 2004; Wand et al., 2017; Verspecht et al., 2019). These approaches have identified the involvement of intrinsically expressed or acquired efflux pumps, such as KpnEF (Abuzaid et al., 2012), CepA (Fang et al., 2002), SmvA (Wand et al., 2017), and AceI (Hassan et al., 2015) as CHX resistance mechanisms. However, based on the membrane disruptive mechanism of action by CHX and its frequent co-association with colistin resistance, there may be other overlapping membrane-specific mechanisms contributing to intrinsic CHX resistance such as lipopolysaccharide (LPS) transcriptional regulators PmrD and PhoPQ as previously identified (Wand et al., 2017). With respect to known colistin resistance mechanisms, many of these regulators are known contributors of LPS alterations that promote colistin resistance (Olaitan et al., 2014).

In this study, we performed an in-depth phenotypic and molecular analysis to identify intrinsic CHX resistance mechanisms using *Escherichia coli* K-12 BW25113 adapted to CHX. We selected this antimicrobial susceptible strain based on its well-established collection of single gene deletions known as the Keio collection (Baba et al., 2006) and gene clone ASKA library collection (Kitagawa et al., 2005). In our study, we gradually adapted *E. coli* BW25113 to CHX over 20 subcultures. We characterized these CHX-adapted *E. coli* isolates for their phenotypic alterations using antimicrobial susceptibility testing (AST) methods to determine antimicrobial cross-resistance, changes in fitness using optical growth curve experiments, scanning electron microscopy (SEM) to identify any cell morphology changes, and a recently published membrane integrity assay to detect membrane alterations. We also conducted in-depth multi-omics analyses of these isolates using whole genome sequencing (WGS), liquid chromatography tandem mass spectrometry (LC-MS/MS) proteomics techniques, and RNA-seq transcriptomic analyses. This combined “omics” approach identified single nucleotide variants (SNVs) in genes in CHX-adapted isolates as well as their associated proteomic and transcript alterations caused by prolonged CHX exposure. Genes with SNVs identified from this analysis potentially contributing to CHX resistance were also examined for their ability to phenotypically complement CHX-adapted and un-adapted isolates using ASKA plasmid library gene clones and in-frame single gene deletions in BW25113. This in-depth multi-omics study revealed the involvement of the outer membrane lipoprotein MlaA, which is a component of the retrograde phospholipid transport Mla system, that serves as an intrinsic CHX resistance contributor. We also identified alterations of other stress response pathways often involving acid regulation. Our analyses show that phospholipid removal is an important mechanism contributing to CHX resistance.

## Results

### Gradual Adaptation of *E. coli* Isolates to Increasing CHX Concentrations Resulted in CHX-Resistant Isolates That Showed No Cross-Resistance to Any Other Antimicrobials Tested

To initiate this study, we first performed a CHX-adaptation experiment of *E. coli* K-12 BW25113 using the gradual antiseptic adaptation approach described in a previous study (Bore et al., 2007). As described in this method, we repeatedly sub-cultured BW25113 (in biological triplicate) in Luria-Bertani (LB) broth containing gradually increasing concentrations of CHX-, starting at a sub-inhibitory CHX concentration 0.4 μg/mL, and the cultures that had most turbid growth were sub-cultured repeatedly into fresh media with increasing stepwise CHX concentrations (Supplementary Table 1). After 20 sub-cultures (20 days) with increasing CHX concentrations, we generated three gradually adapted CHX-resistant isolates (CHXR1-3) capable of growing in the presence of 2.4 μg/mL CHX, which was above the minimum inhibitory concentration (MIC) value for the susceptible un-adapted wild-type BW25113 control (WT; Table 1). Using the broth microdilution AST method, we determined the MIC values of each CHX-resistant isolate to CHX as well as its susceptibility to other representative antimicrobials (Table 2). AST results demonstrated that all three CHX-resistant isolates were only resistant to CHX at 2- to 4-fold higher concentrations when compared to the un-adapted WT (Table 2). Surprisingly, all three CHX-adapted isolates had no increase in resistance to the bisbiguanide antiseptic alexidine (ALX), indicating that our adaptation of *E. coli* to CHX was highly selective and did not confer cross-resistance to another commonly used bisbiguanide antiseptic. CHX-adapted isolates were susceptible to all quaternary ammonium compound (QAC) cationic antiseptics we tested based on their MIC values, as well as to all other antibiotics, including colistin (Table 2). It is notable that CHX-adapted isolates were more susceptible to QACs cetyldimethylethylammonium bromide (CDAB) and cetytrimethylammonium bromide (CTAB) and to the aminoglycoside antibiotic tobramycin when compared to the WT (Table 2). Together, our MIC data indicates that the CHX-adapted *E. coli* we generated had no significant antiseptic cross-resistance or antibiotic cross-resistance in this study. This AST result outcome permitted us to study these isolates in more depth to determine CHX-resistant phenotypes only.

**TABLE 1:** Bacterial *E. coli* strains and plasmids used or generated in this study.

**Strain name**	**Genotype; Description of CHX adaptation**	**Final concentration of CHX used for adaptation (μg/mL)**	**References**
BW25113	F- Δ(araD-araB)567 Δ*lacZ4787*:*rrnB*-3, λ-, *rph-1* Δ(*rhaD-rhaB*)568 *hsdR514*	—	Baba et al., 2006
CHXR1	BW25113; Replicate isolate 1 adapted to CHX after 20 subcultures	2.4	This study
CHXR2	BW25113; Replicate isolate 2 adapted to CHX after 20 subcultures	2.4	This study
CHXR3	BW25113; Replicate isolate 3 adapted to CHX after 20 subcultures	2.4	This study
JW2343-KC	F-, Δ(*araD-araB*)567, Δ*lacZ*4787:*rrnB*-3, λ-, Δ*mlaA*:*kan*, *rph-1*, Δ(*rhaD-rhaB*)568, *hsdR*514	—	Baba et al., 2006
JW4276-KC	F-, Δ(*araD-araB*)567, Δ*lacZ*4787:*rrnB*-3, λ-, Δ*fimE*:, *rph-1*, Δ(*rhaD-rhaB*)568, *hsdR*514	—	Baba et al., 2006
JW5248-KC	F-, Δ(*araD-araB*)567, Δ*lacZ*4787:*rrnB*-3, λ-, Δ*marR*:, *rph-1*, Δ(*rhaD-rhaB*)568, *hsdR*514	—	Baba et al., 2006
JW5490-KC	F-, Δ(*araD-araB*)567, Δ*lacZ*4787:*rrnB*-3, λ-, Δ*yghQ*:, *rph-1*, Δ(*rhaD-rhaB*)568, *hsdR*514	—	Baba et al., 2006
JW3471-AM	F-, Δ(*araD-araB*)567, Δ*lacZ*4787:*rrnB*-3, λ-, Δ*yhiS*:, *rph-1*, Δ(*rhaD-rhaB*)568, *hsdR*514	—	Baba et al., 2006
JW3480-KC	F-, Δ(*araD-araB*)567, Δ*lacZ*4787:*rrnB*-3, λ-, Δ*gadE*:, *rph-1*, Δ(*rhaD-rhaB*)568, *hsdR*514	—	Baba et al., 2006
JW5013-KC	F-, Δ(*araD-araB*)567, Δ*lacZ*4787:*rrnB*-3, λ-, Δ*cdaR*:, *rph-1*, Δ(*rhaD-rhaB*)568, *hsdR*514	—	Baba et al., 2006

**Plasmid name**	**Plasmid details**	**Antimicrobial resistance marker**	**References**

pCA24N(–)	Parental; T5-*lac* promoter expression vector	CM	Kitagawa et al., 2005
pMlaA	pCA24N(–) with *mlaA* cloned with His_6_-affinity tag fusions at the C-terminus	CM	Kitagawa et al., 2005
pYghQ	pCA24N(–) with *yghQ* cloned with His_6_-affinity tag fusions at the C-terminus	CM	Kitagawa et al., 2005
pFimE	pCA24N(–) with *fimE* cloned with His_6_-affinity tag fusions at the C-terminus	CM	Kitagawa et al., 2005
pYhiS	pCA24N(–) with *yhiS* cloned with His_6_-affinity tag fusions at the C-terminus	CM	Kitagawa et al., 2005
pGadE	pCA24N(–) with *gadE* cloned with His_6_-affinity tag fusions at the C-terminus	CM	Kitagawa et al., 2005
pCdaR	pCA24N(–) with *cdaR* cloned with His_6_-affinity tag fusions at the C-terminus	CM	Kitagawa et al., 2005
pMarR	pCA24N(–) with *marR* cloned with His_6_-affinity tag fusions at the C-terminus	CM	Kitagawa et al., 2005

*CM, Chloramphenicol; His_6_, hexahistidine.*

**TABLE 2:** A summary of AST MIC values using broth microdilution for each CHX-adapted *E. coli* isolate in this study.

**Antimicrobial agent tested**	**WT**	**CHXR1**	**CHXR2**	**CHXR3**
ALX	2	2	2	2
BZK	18	18	9	9
CDAB	32	**8**	**8**	**8**
CEF	0.5	0.5	1	0.5
CET	30	30	30	30
CHX	2	**8**	**4**	**4**
CIP	0.25	0.25	0.25	0.25
CM	<7.5	15	15	15
COL	1	1	1	1
CPC	8	8	8	8
CTAB	32	**8**	**8**	**8**
DDAB	8	4	4	4
DOM	8	8	8	8
DOX	8	4	4	**2**
ERY	512	256	512	256
KAN	16	16	16	16
LZD	1024	1024	1024	**512**
MEM	0.03	0.03	0.03	0.03
RMP	512	512	512	512
SMX-TMP	16	16	16	16
TLN	0.25	0.25	0.25	0.25
TOB	16	**4**	**4**	**4**
VAN	256	512	512	256

*Bold values indicate > 2-fold differences in MIC as compared to WT. ALX, alexidine dihydrochloride; BZK, benzalkonium chloride; CAZ, ceftazidime; CDAB, cetyldimethylethylammonium bromide; CET, cetrimide bromide; CHX, chlorhexidine digluconate; CIP, ciprofloxacin; CM, chloramphenicol; COL, colistin; CPC, cetylpyridinium chloride; CTAB, cetyltrimethylammonium bromide; DDAB, dodecyl-dimethylammonium bromide; DOM, domiphen bromide; DOX, doxycycline; ERY, erythromycin; KAN, kanamycin; LZD, linezolid; MEM, meropenem; RMP, rifampicin; SXT-TMP, sulfamethoxazole-trimethoprim; TLN, triclosan; TOB, tobramycin; VAN, vancomycin.*

### CHX-Adapted *E. coli* Isolates Are Phenotypically Stable and Reach Higher Final ODs in LB, DG, and M9 Media

We examined all three CHX-resistant isolates for their CHX phenotypic stability to determine how long these isolates maintained CHX resistance after repeated growth without CHX exposure. This experiment has been performed in previous gradual antiseptic laboratory-based adaptation studies (Gadea et al., 2016, 2017) and verified that CHX phenotypes are stable prior to our multi-omics analyses. To confirm that each CHX-adapted isolate had a stable CHX-resistant phenotype, we sub-cultured each isolate in LB broth without CHX over a 10-day experiment, where we performed CHX AST daily to determine any MIC value changes (Table 3). This CHX phenotype stability testing outcome indicated that there was no significant change in MIC values over the course of 9 days for all three CHXR isolates (Table 3). However, CHXR2 and CHXR3 did show a 2-fold reduction in MIC values on day 10 when compared to MIC values from Days 1–9 (Table 3), though these isolates were still 2-fold more resistant to CHX than the WT. CHXR1 showed the greatest CHX phenotypic stability without CHX selection over the 10-day period, where it occasionally showed higher resistance to CHX (Days 3, 4, and 9; Table 3), suggesting CHX resistance fluctuated within a 2-fold range. Based on this outcome, we selected CHXR1 isolates for further in-depth proteomic and transcriptomic analyses as discussed in further sections.

**TABLE 3:** A summary of MIC values determined for each CHX-adapted isolate after 1–10 days of growth without CHX selection in LB growth medium.

**Isolate/Strain**	**MIC of CHX (μg/mL) after specified days without added CHX selection**									
	**Day 1**	**Day 2**	**Day 3**	**Day 4**	**Day 5**	**Day 6**	**Day 7**	**Day 8**	**Day 9**	**Day 10**
WT	2	2	2	4	4	2	2	2	2	2
CHXR1	8	8	16	16	8	8	8	8	16	8
CHXR2	8	8	8	8	8	8	8	8	8	4-8
CHXR3	8	8	8	8	8	8	8	8	8	4-8

*WT, un-adapted wild-type BW25113; CHXR1, CHX-adapted isolate 1; CHXR2, CHX-adapted isolate 2; CHXR3, CHX-adapted isolate 3 (see Table 1).*

**FIGURE 1:**
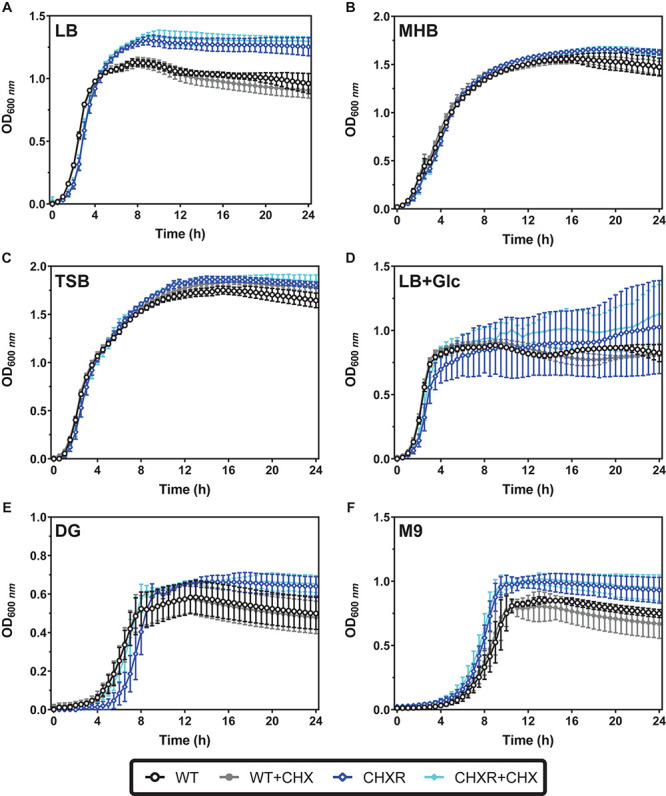
Growth curves of WT and CHXR isolates in rich and minimal media with and without CHX selection. In all panels, growth curves were measured from optical density values at 600 nm (OD_600 *nm*_) in 96-well microtiter plates incubated at 37°C with shaking over 24 h. Averaged values from CHXR1-3 isolates and WT measured in biological triplicate are plotted in each panel, and error bars highlight standard deviation of OD_600 *nm*_ values at each timepoint. In all panels, CHXR isolates are plotted as blue shaded diamonds and WT as circles, where solid filled symbols indicate samples grown in the presence of 0.4 μm/mL CHX final concentration and unfilled symbols indicate media growth without CHX selection. Media with and without CHX addition used to measure the growth of each isolate/strain is shown in each panel: **(A)** LB, **(B)** MHB, **(C)** TSB, **(D)** LB + 0.4% w/v glucose (LB + Glc), **(E)** DG, and **(F)** M9.

The growth phenotypes of each CHX-adapted isolate were also compared to the un-adapted WT using broth optical density (600 nm) growth curve experiments. We compared CHX-adapted isolate growth in various rich media, LB, LB + 0.4% w/v glucose (LB + Glc), Mueller Hinton broth (MHB), and tryptic soy broth (TSB) as well as minimal media, minimal 9 salts (M9), and Davis Glucose (DG). All growth experiments were performed with and without added CHX (at a final 0.4 μg/mL concentration) over a 24 h period at 37°C. Growth curve results helped assess if there were any significant growth delays or reductions for CHXR isolates in specific media and used to identify any changes in the overall fitness of the isolates from lag to stationary phase. To verify final OD values, final colony-forming units/mL (CFU/mL) were determined for diluted (10^–6^ and 10^–7^) 24 h cultures from plated LB agar colonies grown at 37°C. When CHX-adapted isolates were grown without CHX selection in various rich media their final OD_600 *nm*_ values were significantly differed from the WT growth over 24 h in LB, MHB, and TSB (*P*-values of < 0.05) but not LB + Glc (Figures 1A–D, Supplementary Figure 1, and Supplementary Table 2) and grew similar to or slower than WT by 11.6–22.8% differences (Supplementary Table 3). In LB medium, independent of CHX, CHXR isolates reached significantly higher stationary phase OD_600 *nm*_ values after 5 h, ranging from 1.21 OD_600__*nm*_ (±0.07 units error) to 1.37 OD_600 *nm*_ (±0.12 units error) than the WT (1.09 ± 0.03 to 1.11 ± 0.09; *P*-values < 0.05) and corresponded to higher CHXR CFU/mL counts (Supplementary Table 3). CHXR isolate growth in all rich media with CHX selection identified significant changes in final OD_600 *nm*_ values and CFU/mL cell counts when compared to WT under the same conditions in LB, LB + Glc, and MHB (*P*-values < 0.05) but not TSB (Figures 1A–D, Supplementary Figure 1, and Supplementary Table 2, 3). In rich media with CHX selection, CHXR isolates had similar or slower growth doubling times (6.5–24.1% differences) than the WT (Supplementary Table 3). As LB was the medium used to gradually adapt all *E. coli* isolates to CHX for this study, these growth differences may reflect the isolate’s adaptation not only to CHX but also the LB medium components specific to LB formulations.

Growth curves in either minimal media (DG and M9) we tested, showed lower maximum final OD_600 *nm*_ values for all CHXR and WT isolates as compared to their growth in rich media after 12 h (CHXR; 0.61–0.75 OD_600 *nm*_, WT; 0.52–0.58 OD_600 *nm*_), indicating that growth in defined media for all CHXR isolates and WT was reduced (Figures 1E,F, Supplementary Figure 2, and Supplementary Table 3). In minimal media without CHX, final OD_600 *nm*_ values and CFU/mL values for CHXR isolates compared to WT were only statistically different in M9 (*P*-value < 0.05) but not in DG medium (Figures 1E,F, Supplementary Figure 2, and Supplementary Tables 2, 3) suggesting these different defined media formulations influenced CHXR growth rates. In M9 without CHX, CHXR isolates had a mean doubling time that was 33.6% slower than WT but in the presence of CHX, CHXR isolates grew 17.0% faster than WT, indicating that CHX enhances CHXR growth rates (Supplementary Table 3). In DG without CHX addition, CHXR isolates grew faster than WT by 27.2% and in the presence of CHX, CHXR isolates had a mean doubling time that was 9.3% faster than WT (Supplementary Table 3). This shows that CHXR growth rates were faster in minimal media with CHX in contrast to the generally slower CHXR isolate growth rates in rich media. In minimal medium without CHX, CHXR isolates had a slightly longer lag phase of 1 h when compared to CHXR grown in the presence of CHX (Figure 1E and Supplementary Figure 2), however, when we compared final OD_600 *nm*_ and CFU/mL values from CHXR growth curves with and without added CHX, neither was statistically different (Supplementary Tables 2, 3). These findings suggest that prolonged adaptation to CHX measurably alters their growth fitness, where CHXR isolates grew slower than the un-adapted WT in nearly all rich media with and without CHX, whereas when CHX is added to either minimal medium tested, CHXR isolate growth rate was faster than the WT (Figure 1, Supplementary Figures 1, 2, and Supplementary Tables 2, 3). Growth curve findings for specific media conditions (LB + Glc, DG, and M9) also demonstrated that CHXR isolates were capable of growing to higher final OD_600 *nm*_ values and CFU/mL than WT, suggesting some growth conditions maintained or enhanced CHXR isolate fitness.

### CHX-Adapted *E. coli* Isolates Have Altered Cell Morphologies, Where Cells Are Shorter and Narrower Than WT and Have More Permeable Membranes Than WT

**FIGURE 2:**
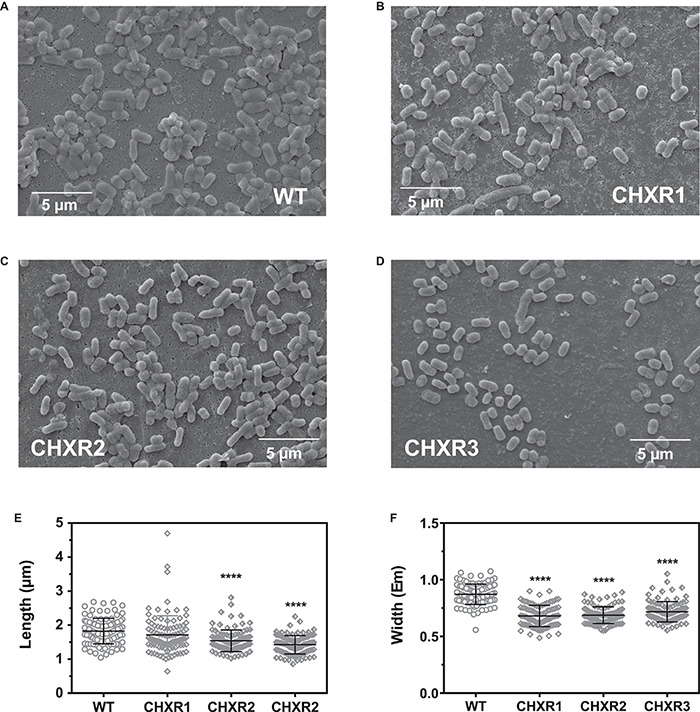
Scanning electron microscopy (SEM) images of CHXR isolates and a summary of measured CHXR cell lengths and widths. **(A–D)** Show representative 5,000X magnification SEM images of WT **(A)**, CHXR1 **(B)**, CHXR2 **(C)**, and CHXR3 **(D)** cells. Bars shown in each panel indicate a 5 μm length as a size reference. **(E,F)** Indicate a summary cell lengths **(E)** and widths **(F)** in μm of 200 cells (*n* = 200) and are shown as plotted symbols. Each CHXR isolate or WT was measured from five SEM images collected from each biological replicate using ImageJ software v1.8.0 measurement tools. Bar plots overlaid on measured cells indicates the maximum, median, and minimum interquartile ranges of the cell measurement datasets and asterisks (****) indicate significant differences between the WT and each CHXR isolate median values at *P*-values < 0.0001.

Our final phenotypic analysis of each CHX-adapted isolate sought to identify if there were any significant alterations in cell morphology with the use of SEM and a novel membrane integrity assay that we had previously published. With SEM imagery, we assessed CHX-adapted isolates grown to mid-log phase and compared them to mid-log WT cell preparations as shown in Figures 2A–D. Based on a visual assessment, all CHX-adapted isolate’s cell surface morphology appeared to be similar to the un-adapted WT, where all cells had the characteristic bacilliform shape (Figures 2A–D). A blinded cell image analysis with ImageJ (Abràmoff et al., 2007) was used determine the mean lengths and widths of 200 cells from two biological replicates of each CHXR and WT cell preparations (Figures 2E,F and Supplementary Figure 3). The analysis revealed that CHXR2 and CHXR3 isolates were significantly (*P* < 0.001) shorter in length than WT cells (WT; 1.82 ± 0.06 μm, CHXR2; 1.75 ± 0.35 μm, CHXR3; 1.42 ± 0.29 μm, Figure 2E and Supplementary Figure 3) and all three CHX-adapted isolates were significantly (*P* < 0.001) narrower in width (CHXR1; 0.68 ± 0.09 μm, CHXR2; 0.69 ± 0.09 μm, CHXR3; 0.71 ± 0.06 μm) than the WT (0.88 ± 0.08 μm) on average (Figure 2F and Supplementary Figure 3). These findings show that CHX adaptation alters cell morphology, by narrowing cell widths overall and in 2/3 isolates, reducing average cell length.

To verify if CHXR isolate membranes were significantly altered from the WT, we examined membrane permeation differences using the membrane impermeant fluorescent dye (propidium iodide). This dye increases in fluorescent emission (at 620 nm) when bound to DNA/RNA and was used to indirectly compare the membrane integrity differences of CHXR1 and WT cells. This commonly used dye is applied to measure dead bacterial cells (Stiefel et al., 2015), and we recently showed it can be used to discriminate the membrane integrity differences between antiseptic-resistant and susceptible Enterobacterial isolates (Gregorchuk et al., 2020). We monitored the relative fluorescent emission unit (RFUs) values of live and heat-treated CHXR isolates and WT cells exposed to the same concentration of dye in buffer after 30-min of exposure. We expected heat-treated (dead) cells would have the highest dye RFUs, as compared to live cells, and live cells with higher cell permeability should correspond to greater dye penetration due to more permeable cell membranes as we verified in Supplementary Figure 4A. A comparison of live mid-log CHXR1 to WT cell preparations showed significantly higher dye RFUs after 30-min for CHXR isolates (2.2-fold increase; mean RFU = 565 ± 248 error, *n* = 9) as compared to WT cells (mean RFU = 252 ± 122 error, *n* = 9; Supplementary Figure 4B). These findings show that CHXR cell membranes are more CHX-permeant than WT and suggests that CHX adaptation alters the *E. coli* membrane from WT, in agreement with our SEM images. Together, our SEM and 30-min impermeant fluorescent dye RFU analyses confirm that CHX adaptation of *E. coli* BW25113 causes phenotypic alterations that can be detected by visual as well as fluorescent techniques.

### Proteomic Analysis of CHX-Adapted *E. coli* Identifies Alterations of Acid Resistance and Stress Response Systems and a Lack of MlaA Protein

**FIGURE 3:**
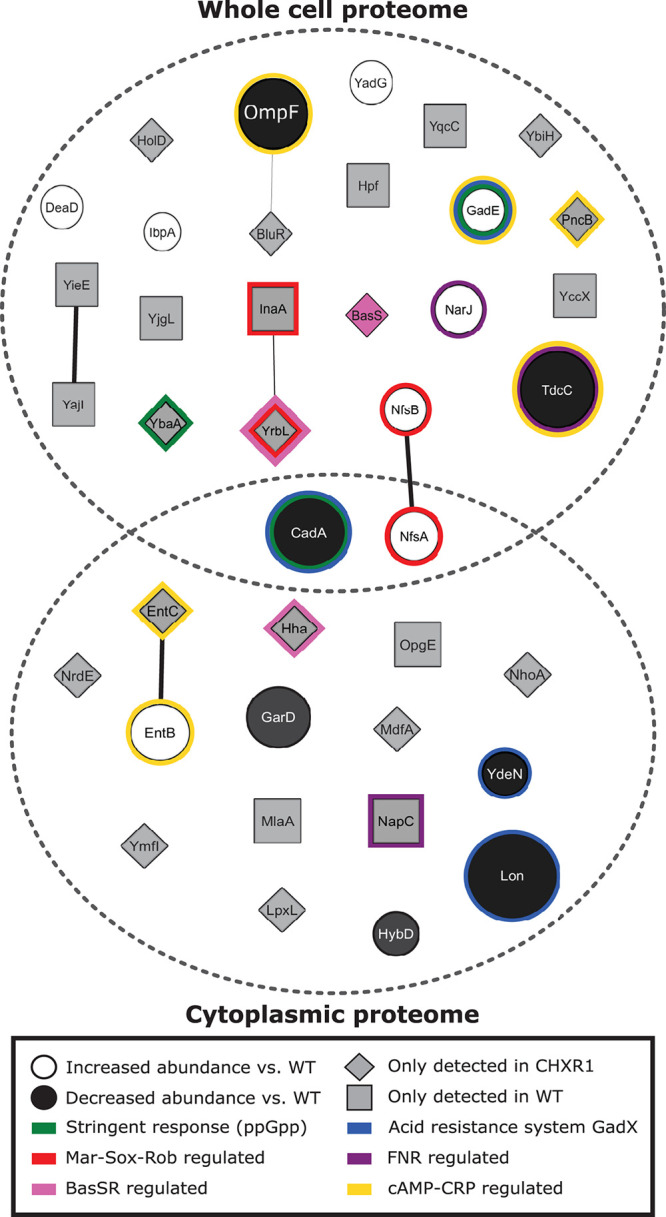
A network diagram summary of significantly identified proteins determined from the CHXR1 isolate and WT proteomic analyses. The network diagram was generated using Cytoscape v3.7.2 (Shannon et al., 2003) using the StringApp v1.5.0 software package (Doncheva et al., 2019). As summarized in the panel legend, significantly altered protein abundances are represented as circles with protein names and indicate proteins that differed between WT and CHXR1 in both the WC and CY proteomes. The size of the protein circles represents the degree of fold change from the WT proteome, where white filled circles represent CHXR1 proteins with increased abundance and black filled circles represent CHXR1 proteins with decreased abundance. Gray filled squares and diamonds represent WT and CHXR1 proteins respectively, that were not detected in the opposite (WT or CHXR1) proteome. Known regulatory systems influencing protein accumulation are outlined by color according to the bottom panel legend.

To identify any alterations in protein presence and abundance between CHXR isolates and WT, we performed proteomic analysis on CHXR1 and WT. We specifically focused on CHXR1 for proteomic analysis due to its greater phenotypic CHX-resistant stability and its higher resistance to CHX (Tables 2, 3). A comparison of CHXR1 and WT whole cell extracted proteomes to cytoplasmic extracted proteomes was conducted using nano LC-MS/MS to determine the relative abundances of soluble and membrane proteins that were altered in the CHXR1 isolate (Supplementary Tables 4, 5). From this analysis, we identified a total of 1904 whole cell (WC) extracted proteins and 2307 cytoplasmic (CY) CHXR1 proteins, of which only 24 WC and 17 CY proteins were noted to significantly differ in abundance or detection when compared to the WT (Figure 3 and Supplementary Tables 4, 5).

Few significantly altered relative protein abundance differences were observed between CHXR1 and WT proteomes (shown as circles in Figure 3), where only two proteins, CadA and NfsA, were differentially accumulated in both the WC and CY extracted proteomes. Lysine decarboxylase (CadA), part of the lysine-dependent organic acid resistance system (Kanjee and Houry, 2013), was decreased in abundance (3.8-fold reduction) in both CHXR1 proteomes as compared to WT. GadE, a central transcriptional activator of glutamic acid decarboxylase (GAD) system that maintains pH homeostasis and regulates multidrug efflux pump MdtEF expression (Ma et al., 2003; Hommais et al., 2004), showed increased (4.6-fold) abundance in the CHXR1 WC proteome (Figure 3). Since CHX is cationic at lower acidity, it was not surprising to see upregulation of pH homeostasis systems in the CHX-adapted proteome. NfsA had significantly increased (2.0-fold) in abundance in the CHXR1 WC and CY proteomes as well as NfsB (1.8-fold) in the WC proteome (Figure 3). NfsA is an NADPH-dependent nitroreductase that catalyzes the reduction of nitrocompounds and is frequently upregulated in *E. coli* exposed to QACs, paraquat, and nitrofuran antimicrobials (Zenno et al., 1996; Whiteway et al., 1998). The increased abundance of NfsA and NfsB in the CHXR1 proteome suggests that this nitrogen reductive pathway may help counteract exposure to toxic nitrogen rich compounds like CHX. Besides NfsAB, many other proteins with altered in abundance between the CHXR and WT were noted to be regulated by stress inducible pathways associated with antimicrobial resistance, such as the Mar-Sox-Rob regulon (Chubiz et al., 2012), the stringent response (Strugeon et al., 2016), and the fumarate and nitrate reduction (FNR) pathways (Kurabayashi et al., 2017). Lon protein, an ATP-dependent protease that degrades misfolded proteins involved in transient multidrug resistance, particularly MarA, SoxS, and GadE (Heuveling et al., 2008; Nicoloff and Andersson, 2013) was significantly reduced (5.3-fold) in abundance in the CHXR1 CY proteomes only (Figure 3). The reduction of Lon in CHXR1 is expected if prolonged activation of the Mar-Sox-Rob and organic acid resistance systems are induced by acidic CHX exposure.

Lastly, nearly half of all proteins we identified in WC and CY proteomes shown in Figure 3 were detectable in either CHXR1 or WT samples (gray filled proteins). Although we cannot compare the relative abundances of these proteins, their detection in the CHX-adapted isolate or WT only were important to note. There was a noticeable absence of MlaA detection in both CHXR1 proteomes (Figure 3), we expected this outcome based on our WGS SNV findings of *mlaA* (Table 4). We also detected MdfA in the in the CHXR1 WC proteome (Figure 3); MdfA is an alkali-resistant chloramphenicol selective multidrug efflux pump belonging to the Major Facilitator Superfamily (MFS) (Edgar and Bibi, 1997; Bohn and Bouloc, 1998). Altered detection of this MFS alkali-resistant efflux pump in CHXR1 suggests upregulated expression of this efflux pump, possibly contributing to CHX-resistance. BasS, also known as PmrB, was detectable only in CHXR1 WC proteomes and is the sensor histidine kinase of the BasSR two-component system that regulates the expression of the *arnBCADTEF* operon which modifies LPS and is often detected in colistin resistant species (Olaitan et al., 2014). Additionally, lipid A biosynthesis enzyme, lauroyl acyltransferase (LpxL) was only detected in the CY proteome of CHXR1, suggesting that LPS biosynthesis may be altered in the CHX-adapted isolate.

**TABLE 4:** A summary of repetitive coding and non-coding SNVs identified from CHX-adapted genomes sequenced in this study.

Coding SNVs*						
**Gene**	**Isolate**	Type of SNV**	**Function**	**Location**	**Locus Tag**	**Uniprot ID^*a*^**
*mlaA/vacJ*	CHXR1	1NS, 1A	Intermembrane phospholipid transport system; outer membrane lipoprotein	Outer membrane	b2346	P76506
	CHXR2	6NS, 1T				
	CHXR3	4NS, 1T				
*yghQ*	CHXR1	10NS, 2F, 2S	Putative multidrug/oligosaccharidyl-lipid/polysaccharide (MOP) flippase superfamily transporter	Plasma membrane	b2983	Q46841
	CHXR3	2NS, 1S				
*yhiS-2*	CHXR2	1S, 1F, 3NS	Pseudogene; putative uncharacterized protein	Unknown	b3504	P37635
*yhiS-1*	CHXR3	7NS				

**Non-coding upstream region SNVs**						

**Gene**	**Isolate**	Type of SNV**	**Function**	**Location**	**Locus tag**	**Uniprot ID**

*mlaA*	CHXR1	12	Intermembrane phospholipid transport system; outer membrane lipoprotein	Outer membrane	b2346	P76506
*insH1*	CHXR2	9	CP4-6 prophage; insertion sequence 5 (IS5) transposase and trans-activator	Plasma membrane	b0259	P0CE49
	CHXR3	2				
*fimE*	CHXR1	11	Transcriptional regulator of fimbria type 1 *fimA*	Cytosol	b4313	P0ADH7
	CHXR2	11				
	CHXR3	16				

*A detailed list of SNVs and their locations is provided in Supplementary Table 2. **The total number of SNVs is provided as well as their type and they are abbreviated as follows: synonymous (S), non-synonymous (NS), frameshifted (F), truncation due to stop codon (T), altered start codon (A). In some cases, the corresponding NS amino acid alteration is highlighted according to its translated residue number. ^*a*^ID refers to Uniprot identification number.

Overall, CHX-adaptation resulted in the significant alteration of a small collection of proteins pivotal in acid resistance, prolonged antimicrobial stress, as well as LPS biosynthesis and transport highlighting many new and previously identified proteins involved in antimicrobial resistance that overlap with CHX-resistance.

### Transcriptomic Analysis of CHX-Adapted Isolates Identifies Reductions in *mlaA* and Upregulation of Acid Regulated Genes

**FIGURE 4:**
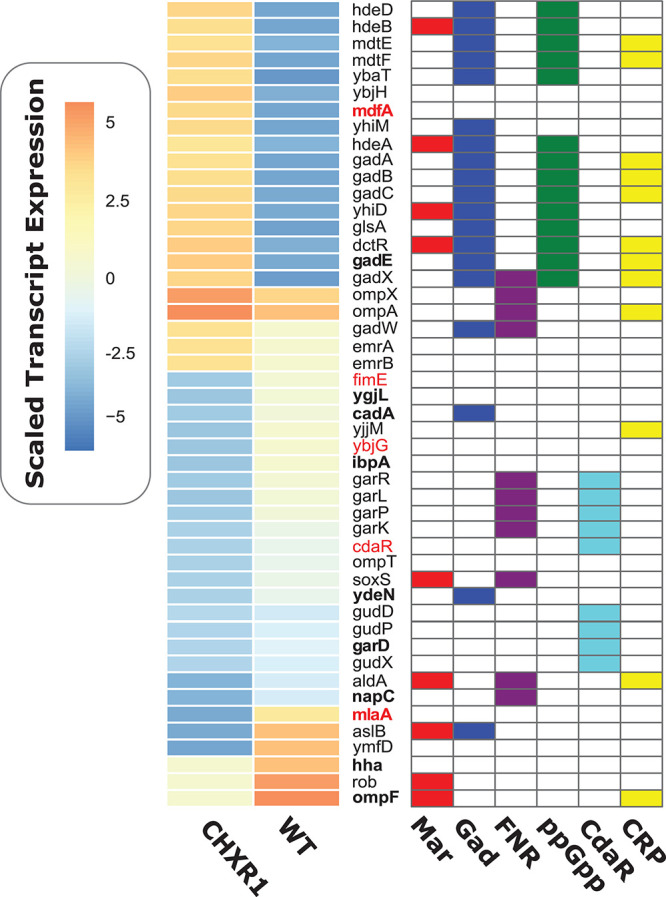
A heatmap diagram of significantly altered gene transcripts of CHXR1 and WT *E. coli* for RNA-seq transcriptional analysis. The heatmap shown to the left of the gene names listed indicates scaled median gene expression values of CHXR1 and WT, where red to orange colors indicate genes that were up-regulated and blue to dark blue colors indicate down-regulated transcripts of CHXR1 to WT. Heat mapped expression data represents median gene expression values from three biological replicates (*n* = 3) of WT and CHXR1 isolates. Colored charts on the right-hand grid beside each *y*-axis gene name indicate whether or not the listed gene is regulated by the mar-sox-rob regulon (Mar; red), GadE/W/X (Gad; blue), fumarate and nitrate reductase regulon (FNR; purple), stringent response system (ppGpp; green), carbohydrate diacid regulator CdaR (light blue), and cyclic AMP-CRP regulon (CRP; yellow). Gene names in red font indicate genes that possessed SNVs (Figure 5B and Table 4) and genes with bolded font indicate translated protein products that were significantly altered in abundance based on proteomic analyses (Figure 3).

To determine if any CHX-adaptation resulted in any gene expression changes in addition to the proteomic changes we observed, RNA-seq transcriptome analyses was performed on mid-log cultures of CHXR1 and WT under the same growth conditions used for proteomic analyses. A total of 4490 genes had altered gene expression as detected by RNA-seq analysis, where only 505 were significantly up- or down-regulated in CHXR1 and WT transcripts (Supplementary Table 6). In an effort to identify significantly altered transcripts from other omics analyses performed herein (proteomic and genomic), we generated a summary heatmap of these differentially expressed gene from CHXR1 and WT (Figure 4). This analysis identified that 11 CHXR1 transcripts up or down regulated from RNA-seq were also similarly altered in protein abundance (bolded genes; Figure 4). Notably, up-regulated efflux pump *mdfA* and down-regulated porin *ompF* transcripts in CHXR1 (Figure 4), had similar accumulation differences in their translated CHXR1 proteins when compared to WT proteomes (Figure 3). In CHXR1 transcriptomes, efflux pump *emrAB* was up-regulated 2-fold, whereas only three additional porins *ompX* (3.1-fold up-regulated), *ompA* (2.2-fold upregulated) and *ompT* (4.3-fold down-regulated) exhibited altered expression in CHXR1 when compared to WT (Figure 4). Acid resistance and pH regulatory genes *cadA* and *gadE* previously identified as having altered protein accumulation also demonstrated similar alterations in transcript levels in CHXR1 transcriptomes (Figure 4). Up-regulation of *gadE* transcripts (8.1-fold from WT) and other GAD system transcriptional regulators *gadX* (4-fold), and *gadW* (2-fold) were detected in CHXR1 when compared to the WT transcriptome (Figure 4). Altered expression of GAD regulated genes were also noted and included *mdtEF*, *gadABC*, *cadA*, *hdeABD*, and *ydeN* (Mates et al., 2007) when compared to the WT transcriptome (Figure 4); YdeN is important to note as it was also down-regulated in CHXR1 proteomes (Figure 3). This suggests that *gadE* and the GAD system regulon may play a role in *E. coli* CHX resistance.

Altered expression of genes in CHXR1 that were genetically altered by SNVs based on WGS were identified for *mlaA* and the carbohydrate diacid regulator *cdaR* (Table 4). Reduced transcript levels of outer membrane lipoprotein *mlaA* (6.4-fold reduction) was identified in CHXR1 and this finding also corresponds with proteomic data where MlaA protein was only detectable in WT CY proteomes only, highlighting its consistent absence in CHXR isolates. The carbohydrate diacid regulator *cdaR*, which had a single frame shift mutation in *cdaR* in the CHXR1 genome, was also significantly down-regulated (6-fold) in the CHXR1 transcriptome (Figure 4 and Supplementary Table 6). Many of the genes regulated by *cdaR*, such as D-glucarate degrading enzymes *gudDPX* and D-galactarate degrading enzymes *garDKPLR* (Monterrubio et al., 2000), were noticeably down-regulated in CHXR1 as well (Figure 4 and Supplementary Table 6), suggesting that the reduction of each carbohydrate degrading enzyme pathway may increase these osmolytes and contribute to CHXR1 resistance.

Lastly, antimicrobial stress inducible genes *rob* and *soxS* were down-regulated 3 to 4-fold in CHXR1 (Figure 4). *rob* and *soxS* are part of the multiple antibiotic resistance *mar-sox-rob* regulon that directly regulates efflux pumps (*acrAB-tolC*) and indirectly *ompF* porin transcription (via *micF*) (Chubiz and Rao, 2011), however, we only detected significant *ompF* down-regulation in CHXR1 (Figure 4 and Supplementary Table 6). Altogether, this indicates that antimicrobial, and pH/acid inducible stress systems are significantly altered in CHX-adapted *E. coli*, in addition to genes corresponding to antiseptic resistance mechanisms (i.e., efflux pump, LPS modifiers, and porins).

### WGS of CHX-Adapted *E. coli* Isolates Identifies Repetitive Deleterious SNVs in *mlaA* Gene in All Three Isolates

**FIGURE 5:**
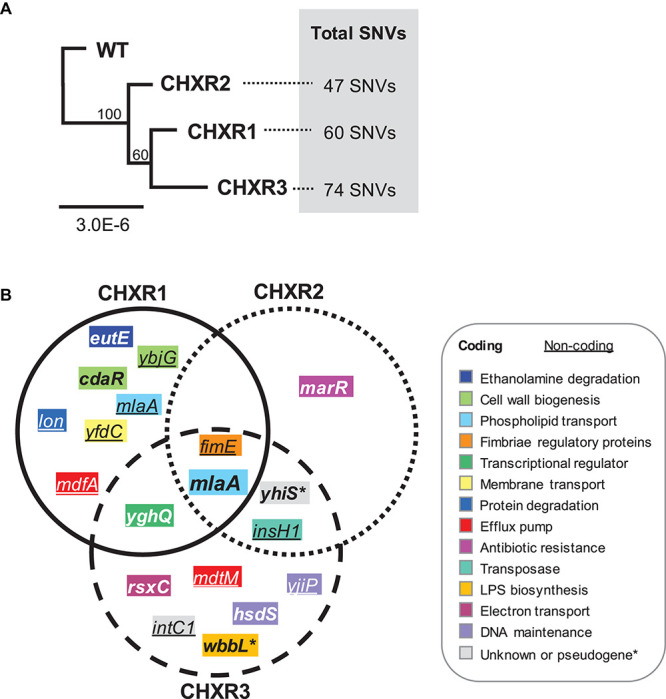
Phylogenetic analysis of CHXR isolate genomes and a Venn diagram summary of coding and non-coding SNVs identified in each CHXR isolate. **(A)** A Maximum Likelihood dendrogram of aligned WGS WT and CHXR1-3 sequences, where bootstrap confidence values are indicated at each node. Total SNVs identified from genomic comparisons of each CHXR isolate to the WT is indicated on the right-hand side of the dendrogram. **(B)** A Venn diagram summary highlighting SNVs identified in the coding genes and non-coding upstream regions of genes (underlined) identified from CHXR isolates by WGS. The gene names shown in the diagram are color-coded according to their functional association as shown in the panel legend.

To discern the genetic consequences of CHX-adaptation, we performed WGS analysis at 30X coverage on each CHX-adapted isolate (CHXR1-3) and compared them to sequenced WT as summarized in Supplementary Tables 7, 8. Findings from this analysis identified relatively few SNVs among each CHXR isolate (47–74 SNVs total), indicating that prolonged CHX-exposure did not generate numerous SNVs in this *E. coli* strain (Figure 5A and Supplementary Table 8). Maximum Likelihood phylogenetic analyses of the sequenced genomes showed that all CHX-adapted isolates grouped together and separately from the WT sequence, where CHXR1 and CHXR3 grouped more closely together than CHXR2 but at lower bootstrap confidence values (60/100; Figure 5A). After comparing CHXR genomic sequences to the sequenced WT reference we identified SNVs in a number of coding and non-coding regions as summarized in Supplementary Table 8. However, very few of these SNVs were repeatedly identified in the same gene(s) amongst all three CHXR isolates as summarized in Figure 5B and Table 4. As shown in the Venn diagram SNV summary of Figure 5B only a single gene *mlaA* (also known as *vacJ*), encoding for an outer membrane lipoprotein and component of the Mla retrograde phospshoplipid transport system, was repeatedly identified in all three CHXR isolates. In each CHXR isolate, the *mlaA* gene possessed deleterious SNVs that either truncated the gene, resulting in reading frameshifts (CHXR2-3) or eliminated its start codon (CHXR1) as noted in Table 4. Several SNVs were also repeatedly identified in 2/3 CHXR isolates, specifically, in *yghQ* a putative multidrug/oligosaccharidyl-lipid/polysaccharide (MOP) flippase superfamily transporter (Saier et al., 2016) and a pseudogene *yhiS* (*yhiS*-1, *yhiS*-2) which is disrupted by an insertion element and encodes for an as yet uncharacterized protein (Figure 5B and Table 4).

Our analysis of non-coding SNVs in repetitively occurring regions of the CHX-adapted isolates primarily focused on upstream genetic regions for their potential to disrupt coding sequence expression/regulation (Table 4). In all three CHXR isolates, there was only one set of repetitively identified non-coding SNVs in the upstream of *fimE*, which is a transcriptional regulator of the type 1 fimbrial gene *fimA.* FimE is a recombinase that controls the “ON-OFF” *fim*-switch (*fimS*) that regulates transcription of the type 1 fimbrial operon referred to as phase-variation (Kulasekara and Blomfield, 1999). In CHXR1 transcriptomes, *fimE* was down-regulated by 3.6-fold but no significant changes in any other *fim* operon gene expression was identified (Figure 4 and Supplementary Table 6). More in-depth analysis of the *fimS* region from WGS contigs revealed that all three CHXR possessed SNVs and indel alterations of the *fimS* resembling a hybrid ON-OFF promoter sequence (Supplementary Figure 5), which may explain why *fimE* expression was reduced in CHXR1 when compared to WT. The only other repetitively identified non-coding SNV region was in the upstream region of *insH1* in CHXR2-3 isolates. InsH1 is a transposase and transcriptional trans-activator of the insertion sequence element IS5 and it is a stress inducible gene within the cryptic prophage (CP) 4–6 in *E. coli* K-12 (Schnetz and Rak, 1992). Transcripts of *insH1* were not shown to significantly differ between CHXR1 and WT transcriptomes (Supplementary Table 6). Lastly, it is worth noting that the upstream region of *mlaA* in the CHXR1 isolate had numerous nucleotide sequence substitutions and transversions that altered the putative –35 and –10 regions of the *mlaA* promoter (Supplementary Table 8). When this finding is combined with coding SNV data showing the start codon of *mlaA* is altered in CHXR1, all three CHXR isolates are expected to have non-functional *mla* genes. In summary, WGS revealed that relatively few genes were repetitively altered in all CHX-adapted and suggests the involvement of *mlaA* and *fimE* in all CHXR isolates.

### Complementation of CHXR Isolates With *mlaA* Reverts the CHX-Resistant Phenotype to WT

**FIGURE 6:**
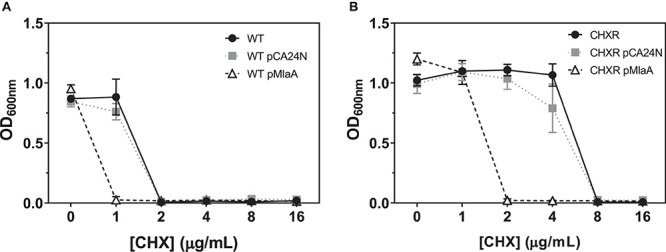
A summary of broth microdilution AST OD_600 *nm*_ values for CHXR and WT pCA24N and pMlaA transformants after 18 h of growth at 37°C. **(A)** pCA24N, and pMlaA transformed CHXR1-3 isolates OD_600 *nm*_ values after 18 h of growth at 37°C at increasing CHX concentrations. The results of CHXR1-3 isolate OD_600 *nm*_ values are shown as a plotted average and error bars indicate standard deviation. **(B)** pCA24N, and pMlaA transformed WT (BW25113) isolates OD_600 *nm*_ values after 18 h of growth at 37°C at increasing CHX concentrations with chloramphenicol selection. Untransformed CHXR1-3 isolate **(A)** or WT **(B)** OD_600 *nm*_ values are shown in each panel as a negative control reference and were grown in the absence of chloramphenicol. All transformant AST OD_600 *nm*_ values were measured in biological triplicate.

Our final aim sought to identify which genes that were confidently and repeatedly identified in our multi-omics analyses of CHX-adapted isolates, specifically contributed to overall CHX-resistance mechanisms. Using the *E. coli* ASKA plasmid clone library we transformed plasmids with cloned genes for *marR, mlaA, yghQ, yhiS, fimE, gadE*, and *cdaR* (Table 1), into each CHXR isolate and the WT strain to determine if there were any significant changes in CHX susceptibility using broth microdilution AST methods as previously described. The results from all plasmid complementations revealed that only CHXR isolates transformed with pMlaA showed significant 4-fold reductions in CHX MIC values as compared to the parental vector pCA24N (Figure 6A and Table 5). Complementation of the WT strain with these plasmids also demonstrated a significant reduction in CHX MIC values for pMlaA only (Figure 6B and Table 5). No changes in CHXR1-3 transformant CHX MIC values were determined for pYghQ, pFimE, or pMarR indicating that *mlaA* primarily influences the CHX-resistant phenotype (Table 5). It is important to note that transformation of WT with pFimE resulted in a 2-fold increase in CHX MIC when compared to the parental vector but no changes in CHX MIC were observed in any CHXR isolate transformed with pFimE (Table 5), indicating that FimE had no impact on CHX-resistant phenotypes. Additionally, only pCdaR CHXR1 transformants showed 2-fold CHX MIC reduction as compared to the parental vector (Table 5). Since CHXR1 was the only isolate to have *cdaR* mutations, the partial complementation of a functional *cdaR* gene suggests this carbohydrate diacid regulator may also contribute to CHX resistance mechanisms. However, *cdaR* likely is most effective in combination with Δ*mlaA* deletions based on the lack of CHX MIC values difference from the WT in Δ*cdaR* strain (Table 5).

**TABLE 5:** A summary of CHX MIC values from AST of plasmid transformed WT, CHXR isolates, and *E. coli* K-12 single gene deletions.

	**Mean transformant CHX MIC values (μg/mL)**								

**Strain/isolate transformed**	**pCA24N**		**pMlaA**	**pYghQ**	**pMarR**	**pFimE**	**pYhiS**	**pCdaR**	**pGadE**
BW25113 (WT)	2		1	2	2	**4**	2	2	2
CHXR1	8		**2**	8	8	8	8	**4**	8
CHXR2	8		**2**	8	8	8	8	8	8
CHXR3	8		**2**	8	8	8	8	8	8

**Deletion strain**		**Mean CHX MIC values (μg/mL)**							

BW25113 (WT)		2							
JW2343-KC (Δ*mlaA*)		**4**							
JW5490-KC (Δ*yghQ*)		**4**							
JW4276-KC (Δ*fimE*)		2							
JW3471-KC (Δ*yhiS*)		2							
JW3480-KC (Δ*gadE*)		**4**							
JW5013-KC (Δ*cdaR*)		2							

*Bold values indicate significant >2-fold MIC changes as compared to the relevant control (pCA24N or WT).*

Antimicrobial susceptibility testing of *E. coli* K-12 BW25113 Keio collection strains containing single gene deletions the same complemented genes listed above, revealed that only the deletion of Δ*mlaA* (JW2343-KC), Δ*yghQ* (JW5490-KC), and Δ*gadE* (JW3480-KC) increased CHX MIC values by 2-fold from the WT (Table 5). This suggests that the loss of *mlaA* in *E. coli*, as well as the individual deletion of putative polysaccharide exporter *yghQ* or the GAD transcriptional regulator *gadE* can also confer modest CHX-resistance enhancements based on MIC values. Growth curve analysis of Δ*mlaA* and WT showed no statistically significant differences in growth rate in all media tested (Supplementary Figure 6 and Supplementary Table 3). However, similar to CHXR isolate growth curves, Δ*mlaA* also had significantly higher final OD_600__*nm*_ values than WT (by 20–29%) in all media tested (LB, MHB, and DG) with and without added CHX (Supplementary Figure 6). These findings confirm that pMlaA complementation and *mlaA* over-expression in CHXR and WT strains is detrimental for CHX resistance and in fact even increases WT cell susceptibility to CHX. Our results also identify additional *E. coli* genes (*yghQ*, *fimE*, *gadE*, and *cdaR*) that when altered, may additively contribute toward CHX-resistant phenotypes and serve as additional CHX biomarkers with *mlaA*.

## Discussion

The findings from this phenotypic and multi-omics study of CHX-adapted *E. coli* isolates have revealed the importance of MlaA in intrinsic CHX resistance. Outer membrane lipoprotein MlaA forms a complex with outer membrane porins OmpF and OmpC (Chong et al., 2015), and serves as the outer membrane component linking the remaining MlaFEDBC system components in the plasma membrane and periplasm to the outer membrane. The Mla system, referred to as the maintenance of outer membrane lipid asymmetry in Gram-negative species, functions as a retrograde phospholipid transport system. This system removes phospholipids from the outer membrane back into the plasma membrane in an ATP-dependent process (Ekiert et al., 2017). Retrograde phospholipid trafficking helps maintain LPS enrichment in the outer leaflet of the outer membrane and assists in cellular phospholipid recycling. In our study of CHX-adapted *E. coli* isolates, we observed deleterious SNVs in a single gene, *mlaA* amongst all three CHXR isolates indicating its involvement in CHX resistance (Figure 5B and Table 4). This suggests that intrinsic CHX resistance by *E. coli* and potentially other Gram-negative species is enhanced when the retrograde phospholipid Mla transport system is inactivated and may be an important contributor to CHX resistance mechanisms. Since CHX acts by forming gaps between paired lipids, we speculate that CHX may be brought along with its paired lipids as they are returned back to the plasma membrane by the Mla system, this may also identify a route of entry of CHX into the cell. When the MlaA is absent, as we observed in our CHX-adapted isolates, CHX entry and potentially its membrane disruptive mechanisms of action are prevented.

Our findings related to MlaA and CHX-resistance herein appear to be novel, however, other Mla system components have been previously identified as antimicrobial resistant contributors in various proteobacterial studies (Bernier et al., 2018; Elliott et al., 2020). MlaC, a periplasmic spanning protein component of the Mla system was previously shown to confer resistance to antimicrobial peptide arenicin-3 in uropathogenic strains of *E. coli* (Elliott et al., 2020). MlaC was also shown to participate in the intrinsic resistance of *Burkholderia cepacia* complex species, where mutants were more susceptible to Gram-positive selective antibiotics (macrolides, rifampin) as well as fluoroquinolones and tetracyclines (Bernier et al., 2018). In our study, CHXR isolates were more susceptible to cationic antiseptic QACs (CTAB and CDAB) and tobramycin which is cationic antibiotic at neutral pH (Table 2). Although we included Gram-positive selective antimicrobials rifampin, vancomycin, and macrolide erythromycin, we did not see any significant changes in MIC values for our CHXR isolates (Table 2). This may suggest that *mlaA* mutants we observed in our *E. coli* isolates may confer more selective antimicrobial resistant profiles as compared to mutations in internal *mlaFEDBC* system inter-membrane spanning components.

Our adaptation of *E. coli* K-12 to CHX did not result in colistin cross-resistant phenotypes as shown in previous studies of *Klebsiella* and *Salmonella* species (Wand et al., 2017; Hashemi et al., 2019; Verspecht et al., 2019). We did, however, identify two proteins (BasS, LpxL; Figure 5B) in our CHXR1 isolate that have been previously implicated as mechanistic contributors of colistin resistance and LPS modification (Olaitan et al., 2014). Based on our study, the alteration of only two proteins previously associated with colistin resistance was insufficient to confer colistin cross-resistance in CHX-adapted *E. coli*. Our findings also show that the LB growth medium we used to initially adapt *E. coli* K-12 only contained CHX, and as result, it generated adapted isolates with resistance exclusively to CHX and even excluded cross-resistance to the other bisbiguanide ALX we tested (Table 2). The chemical structure of ALX differs significantly from CHX as ALX possesses ethylhexane chains instead of benzene rings at the end of the biguanidine moieties. These key chemical differences likely impact how each bisbiguanide displaces or disrupts phospholipid associations and could result in different lipid-to-bisbiguanide compound configurations, thereby affecting different outer membrane systems. ALX was previously shown to have a higher affinity for LPS binding than CHX, which may be an influential factor (Zorko and Jerala, 2008). It is also important to note that species isolated from the environment are likely exposed to far more antimicrobials than the controlled conditions of our study and the lack of antimicrobial cross-resistance we observed in our CHX-adapted isolates may simply reflect controlled lab conditions. Additionally, *E. coli* K-12 lacks the *O*-antigen moiety present in most pathogenic *E. coli* strains and LPS antigens present in other Enterobacterial clinical isolates like *Klebsiella* spp. and *Salmonella* spp. (Rai and Mitchell, 2020). Future studies of *mlaA* and its role in antimicrobial resistance and antiseptic resistance can now focus on exploring other Enterobacterial species including clinical isolates, which may identify additional outer membrane contributors to intrinsic CHX-resistance and cross-resistance.

A notable finding from transcriptome and proteomic analyses show a small, but potentially important role for two acid regulatory systems in CHX resistance, specifically the GAD (*gadE*) system and carbohydrate diacid regulator (*cdaR*) (Figures 3, 4). Due to the cationic charge of CHX in solution (<pH 7), CHX can decrease pH as its concentration increases, likely triggering acid resistance system requirement. The glutamate dependent GAD system transcriptional activator *gadE* had increased protein abundance and transcript levels in CHXR1 multi-omics analyses (Figures 3, 4). GadE, along with GAD system regulators GadX/GadW, regulate the expression of many genes involved in pH homeostasis and acid resistance, including the efflux pump *mdtEF* gene which were shown to be transcriptionally up-regulated in CHXR1 (Figure 4). Complementation of pGadE in all three CHXR isolates did not alter CHX MIC values, however, deletion of Δ*gadE* in *E. coli* did increase WT resistance by 2-fold (Table 5). This suggests gadE deletions may partially contribute to CHX resistance mechanisms. Additionally, carbohydrate diacid regulator CdaR was another proteomically and transcriptomically diminished protein/transcript in CHXR1 (Figures 3, 4), that was also mutated by a single frame shifting SNVs in the CHXR1 isolate (Table 4). As we observed from AST, only pCdaR CHXR1 had a 2-fold reduced CHX MIC value (Table 5), suggesting that the loss CdaR expression can partially rescue the CHX susceptible phenotype back to WT levels. Since CdaR regulates the D-glucarate (*gudPDX*) and D-galactarate (*garPLRK*) uptake and metabolism (Monterrubio et al., 2000), the build-up of glucarate and galactarate compounds may offset the pH and osmotic stress associated with CHX exposure. Although neither *gadE/cdaR* gene deletion or plasmid complementations resulted in significant CHX MIC changes on their own (Table 5), it is likely that *cdaR* deletion or *gadE* up-regulation may be important additive acid regulatory systems with Δ*mlaA* to confer greater intrinsic CHX resistance.

Lastly, our phenotypic analysis of CHX-adapted *E. coli* isolates herein demonstrated that these isolates had similar or enhanced growth rates as the un-adapted WT across the rich and minimal media we tested (Figure 1, Supplementary Figures 1, 2, and Supplementary Tables 2, 3). CHXR cells were also narrower and shorter than WT cells in mid-log phase based on SEM imaging (Figure 2) and more permeant to the dead cell stain propidium iodide (Supplementary Figure 4), indicating that the cell membranes of CHXR1 are more compromised than WT. Molecular explanations for these morphological alterations are likely due to expected outer membrane phospholipid distribution problems that would be caused by the loss of functional MlaA in the CHXR isolates (as discussed above). Our growth curve analyses of WT and (Δ*mlaA*) revealed that the loss of *mlaA* increased CHXR cell CFU/mL numbers and cells grew to higher final ODs than WT but CHXR isolates did not increase doubling times significantly from WT (Supplementary Figure 6 and Supplementary Table 3). We speculate that the lack of phospholipid recycling caused by the deletion of *mlaA* in cells exposed to CHX, helps the cell overcome its membrane disruptive actions by providing more phospholipids for CHX to interact with, ultimately slowing CHX entry into the cell. In addition to *mlaA* mutations, we identified non-coding alterations to the promoter region of the transcriptional regulator *fimE*, which is an important type 1 fimbrial regulator (Figure 3 and Table 4). Although changes in type 1 fimbrial expression primarily impacts cell attachment, previous studies have also shown that type 1 fimbrial gene (*fim*) mutants can influence bacterial colony morphology (Hasman et al., 2000). Our transcriptomic analysis of CHXR1 identified that *fimE* was significantly down regulated in CHXR1 but *fimAICDFGH* operon expression was not significantly altered as compared to WT (Figure 4 and Supplementary Table 6). This might be explained by our WGS findings; all CHXR isolates had significant SNVs and indels that altered the *fimS* promoter region regulated by *fimE*, where it had a hybrid ON-OFF sequence configuration (Supplementary Figure 5). This hybrid promoter may explain the *fimE* and *fimAICDFGH* operon transcriptome results (Figure 4). Transformation of CHXR isolates with plasmids expressing *fimE* failed to alter their CHX MIC values (Table 5) but deletion of *fimE* in *E. coli* (JW4276-KC) did increase CHX resistance by 2-fold as compared to WT (Table 5). This suggests that similar to *gadE* and *cdaR*, *fimE*, and the *fim* operon it transcriptionally regulates may also play another small but additive role when combined with the Δ*mlaA* phenotype.

In conclusion, our phenotypic and multi-omics analyses of CHX-adapted *E. coli* K-12 isolates has identified the involvement of *mlaA* as an intrinsic CHX resistance contributor. The role of MlaA, the outer membrane component of the Mla retrograde phospholipid transport system, in CHX resistance is a novel finding that may have broader implications for intrinsic CHX resistance in *E. coli* as well as other Gram-negative bacterial species. The Mla system component MlaC, was shown to contribute to antimicrobial resistance in other proteobacterial species, such as *Burkholderia* (Bernier et al., 2018) highlighting the importance of this lipid transport system as a potential therapeutic antimicrobial target. When combined with our proteomic and transcriptome analysis, the findings of our study offer greater insights into the mechanism of intrinsic CHX resistance and identify many other pertinent antiseptic resistant biomarkers (*yghQ, fimE, gadE, cdaR*) that may be helpful for future initiatives to rapidly detect CHX-resistant phenotypes.

## Materials and Methods

### Chemicals and Bacterial Strains Used in This Study

Chlorhexidine digluconate (CHX), as well as all other antimicrobials and chemicals used in this study were obtained from either Tokyo Chemical Industry America (United States), Millipore Sigma (United States), Fisher Scientific (United States), or VWR (Canada). *E. coli* K-12 BW25113 (Baba et al., 2006) was obtained from the Coli Genetic Stock Centre (CGSC^3^). All cultures were grown with selection when necessary in Luria-Bertani (LB) broth at 37°C with shaking at 150 revolutions per min (RPM) (WT transformants: 30 μg/mL chloramphenicol (CM); CHXR: 2 μg/mL CHX; CHXR transformants: 2 μg/mL CHX and 30 μg/mL CM).

### CHX Adaptation Experiments

Chlorhexidine adaptation experiments were performed in LB broth medium using a gradual adaptation sub-culturing method as described by Bore et al. (2007) with modifications. Briefly, *E. coli* K-12 BW25113 was initially grown overnight (18 h) from a dimethylsulfoxide (DMSO) cryopreserved stock and then diluted 10^–2^ into 5 mL LB containing 0.4 μg/mL CHX (20% of the WT MIC value) in triplicate. The next day, each individual culture was re-inoculated into 5 mL of fresh LB containing 0.4 μg/mL CHX and grown overnight until day 12 to expose the cultures to prolonged sub-inhibitory CHX. After day 12, cultures were exposed to increasing CHX above the MIC value of WT (2 μg/mL) for an additional 8 generations for a total of 20 days. This process generated three independent isolates: CHXR1, CHXR2, and CHXR3 (Table 1) and a summary of the CHX concentrations added to each sub-culture is shown in Supplementary Table 1. At the end of this process, adapted bacteria were cryopreserved in LB with 16% (v/v) glycerol and stored at –80°C.

### Antimicrobial Susceptibility Testing (AST) to Determine MIC Values

A modified broth microdilution AST method (CLSI, 2012) was used to determine MIC values. Briefly, cryopreserved stocks of WT or CHX-adapted *E. coli* were inoculated overnight without selection, then overnight with selection. Culture turbidity was measured and standardized spectrophotometrically to obtain an optical density (OD_600 *nm*_) of 1.0 unit in LB with a Multiskan Spectrum microplate reader (Thermo Fisher Scientific, United States). The adjusted cultures were diluted 10^–2^ into 96-well microtiter plates containing 2-fold serial dilutions of antimicrobial in LB broth. AST for all isolates was performed in technical triplicate. A total of 22 antimicrobial compounds were included for AST and are summarized in Table 1. For antimicrobials that required solubilization in ethanol (linezolid; LZD, rifampicin; RMP, erythromycin; ERY), or DMSO (alexidine; ALX, trimethoprim-sulfamethoxazole; SXT), control plates containing the same concentration of these solvents only were used to assure the results were due to the antimicrobial. Once inoculated, all microplates were incubated overnight before OD_600 *nm*_ spectrophotometric measurement. Significant increases in MIC were defined as greater than 2-fold MIC changes when compared to the WT.

### Growth Curve and CHX Resistance Stability Testing of CHX-Adapted Isolates

Bacterial fitness was assessed for CHX-adapted isolates, WT (BW25113), and JW2343-KC (Δ*mlaA*) in 96-well microplate broth cultures. Microplate growth curves were set up as described for AST testing, however, CHX concentrations used in these assays were 20% of the WT strain’s MIC value (0.4 μg/mL) to compare isolates grown at identical drug exposures. Growth curves were measured spectrophotometrically over 24 h using a Synergy Neo2 Hybrid Multimode reader (Biotek, United States). Twenty-four hour growth curve experiments were repeated for each replicate tested in a variety of rich media [LB, LB plus 0.4% (w/v) glucose; LB + Glc, cation adjusted Mueller Hinton broth; MHB, Tryptic Soy broth; TSB] and minimal (minimal nine salts; M9, Davis Glucose; DG) media. To calculate the doubling time of the WT, Δ*mlaA*, and CHXR isolates in each media, OD_600 *nm*_ values were blank subtracted and plotted on a semi-log graph; calculated values are provided in Supplementary Table 3. The linear slopes of plotted OD_600 *nm*_ trendlines (*R*^2^ ≥ 0.99) were used to calculate each culture’s doubling time/growth rate in 2-min intervals. Final mean colony-forming units/mL (CFU/mL) were determined from the number of colonies produced from three biological replicates of 100 μl of diluted (10^–7^) *E. coli* 24 h culture plated on LB agar and incubated overnight (18 h) at 37°C (Supplementary Table 3).

The phenotypic stability of each CHX-adapted *E. coli* was assessed by repeated sub-culturing of the CHX-adapted isolates in LB without CHX over 10 days. Each day, AST against CHX was performed as described above. All stability experiments were completed in technical triplicate per isolate (Table 3).

### Scanning Electron Microscopy

To identify morphological anomalies between WT and CHX-adapted *E. coli*, SEM was performed using conditions described by Golding et al. (2016). Briefly, bacterial samples were grown with selection to an OD_600 *nm*_ of 0.5 units, pelleted by microcentrifugation (30 s at 14,000 RPM) and resuspended in PBS. Once resuspended, we modified the protocol by diluting the sample 1:1000 instead of 1:10 as to not overload the filter. Each sample’s filter was washed with increasing concentrations of ethanol before being allowed to dry. Samples were mounted onto a carbon disk, put onto an aluminum stud, and contact between the stud and the filter was established using flash dry silver paint. Samples were subsequently sputtered with gold using a Quorum Q150R S (Quorum Technologies, United Kingdom). Each stud was imaged with the JCM-5700 SEM (JEOL, United States), at 5,000X magnification in five different locations on the filter to help assess overall patterns in cell imaging. Twenty bacterial cell lengths and widths were measured in five separate images for each biological replicate (*n* = 100) where measurements were performed using ImageJ v1.8.0 (Abràmoff et al., 2007). Statistical analysis of measured cell lengths and widths were analyzed using Prism6 v6.0 (Graphpad Software, United States) and significant differences were assessed using the Student’s *t*-test (*P* < 0.001). Two biological replicates of each WT and CHXR1-3 cells were measured by SEM, with no significant difference (*P* > 0.05) between each replicate. Differences between WT and CHXR1-3 in terms of average cell lengths and widths were assessed using a Student’s *t*-test.

### Propidium Iodide Dye Cell Permeation Assays

Propidium iodide assays were performed as described by Gregorchuk et al. (2020) using modified live-and heat-treated cell fluorescent dye emission assays. Briefly, mid-log (OD_600 *nm*_ = 0.5 units) cultures of CHXR1-3 and WT were grown as described for SEM, but these preparations were washed in cold phosphate buffered saline and resuspended to a final OD_600 *nm*_ value of 0.2 units and stored on ice. Samples were divided, where half were heat-treated at 120°C for 20 min (heat-treated) and the other were not (live samples) and added to 96-well fluorescent black-walled microtiter plates containing propidium iodide at 2 μg/ml final concentration. Plates containing heat-treated and live cell preparations of CHXR and WT in biological triplicate and technical triplicate were measured in a fluorescent microplate-reader (Polarstar Optima, BMG labtech, Germany) for 30 min at 37°C taking fluorescent emission measurements at 620 nm every 5 min. RFU values were determined for live as well as heat-treated samples, which were baseline subtracted from emission values from wells lacking cells but containing PBS and dye only. RFU values for each heat-treated and live cell reparations are plotted in Supplementary Figures 4A,B. Statistical analysis was performed using Prism v6.0 (Graphpad Software, United States) software to compare significantly different RFU values between WT and CHXR1 samples using Mann–Whitney tests at *P*-values of < 0.05.

### Whole Genome Sequencing (WGS), Genome Assembly, and SNV Analysis

Genomic DNA (10–30 ng/μL) was isolated from each CHX-adapted replicate using a Purelink Microbiome DNA isolation kit (A29790, Thermo Fisher Scientific, United States) according to manufacturer’s instructions for bacterial culture DNA extraction. Genome sequencing was performed by MicrobesNG^4^ (United Kingdom) with an Illumina-MiSeq system (Illumina Inc., United States) at a minimum of 30X coverage. Sequencing details are found in Supplementary Table 2. Trimmed paired reads were generated and assembled in-house using the MicrobesNG pipeline with *E. coli* BW25113 (CP009273.1) as the mapping reference. Each sequenced CHX-adapted *E. coli* genome sequence is available as BioProject ID PRJNA646979 in NCBI GenBank. SNV analysis was performed using Geneious v11.1.5 software (Biomatters Ltd., New Zealand) to identify SNVs and compare DNA sequences from each CHX-adapted replicate to the reference genome. We controlled for genetic drift of the adapted isolates by eliminating any WT SNVs found in the CHX-adapted isolates. A multiple sequence alignment of genome assemblies was created from the mapped reads, and a Maximum Likelihood tree was constructed using *E. coli* BW25113 (NZ_CP009273) as the root sequence using PhyML v.3.3.20180621 (Guindon et al., 2010). Confidence of tree branching was determined by performing 100 bootstrap replicates and these values are indicated at each node in the dendrogram.

### Proteomic Analysis and Gene Ontology

#### Sample Preparation

Proteomic analysis of the CHXR1 was performed on biological triplicate cultures grown in 4 L batches with CHX selection. Cultures were grown to a final OD_600 *nm*_ = 0.5 units and were then harvested by centrifugation at 6000 RPM for 10 min in Avanti-J-E High performance centrifuge (HPC; Beckman, United States). The pellet was resuspended by wet weight in an equal volume of isolation buffer: 50 mM 3-(*N*-morpholino) propane sulfonic acid (MOPS), 8% v/v glycerol, 5 mM ethylenediaminetetraacetic acid (EDTA), 1 mM dithiothreitol, pH 7 and WC pellets were stored frozen at –80°C. Cytoplasmic protein extractions were performed from thawed WC pellet preparations, where pellets were thawed and a final concentration of 0.1 mM phenylmethylsulfonyl fluoride was added. This cell slurry was immediately French pressed in a 4°C chilled Thermofisher Sim Aminco 30,000 lb/in^2^ cylinder twice at 1,500 lb/in^2^ each pressing. The pressed slurry was centrifuged at 10,000 RPM in a JA-20 Beckman rotor, where the supernatant was collected and ultracentrifuged at 40,000 RPM for 90 min in a Type 70Ti rotor in a Beckman Optima XPN-100 ultracentrifuge. The supernatant was saved as the cytosolic protein fraction and protein concentrations were determined using a modified Lowry assay (Shen et al., 2013). This cytoplasmic protein extraction was stored at −80°C.

Thawed WC pellets and cytoplasmic extracted protein samples were prepared similarly as detailed below. Thawed pellets were homogenized in sterile Milli-Q water, mixed with 100 μl 0.1-mm glass beads (Scientific Industries Inc., United States), and heated for 5 min at 95°C. Cells were lysed by highspeed vortex-mixing for 3 min, followed by centrifugation at 3000 RPM for 1 min. The supernatant was collected, and another aliquot of cold sterile Milli-Q water was added to the beads, followed by 1 min of vortexing and 1 min of 3000 RPM centrifugation to pool protein supernatants from the beads. This was repeated five times total to extract the remaining proteins, which was stored at –80°C. Protein was quantified using a bicinchoninic acid (BCA) protein assay kit, with bovine serum albumin (BSA) as the standard (Pierce Protein Research Products; Thermo Fisher Scientific, United States). Hundred microgram of protein was isolated from WT and CHXR1 in biological triplicate, and digested with trypsin (Promega, United States) overnight (16–18 h) using a filter-assisted sample preparation (FASP) method described previously (Wiśniewski et al., 2009). Following digestion, all samples were dried down and reconstituted using mass spectrometry grade water to a final concentration of 1 μg/μl for LC-MS/MS analysis.

#### Nanoflow-LC-MS/MS

Each sample was separately analyzed using a nano-flow Easy nLC 1200 connected in-line to an Orbitrap Fusion Lumos mass spectrometer with a nanoelectrospray ion source at 2.3 kV (Thermo Fisher Scientific, United States). The peptide samples were loaded (2 μl) onto a C_18_-reversed phase Acclaim PepMap 100 trap column (2 cm × 75 μm, 3 μm particles; Thermo Fisher Scientific, United States) with 30 μL of buffer A (2% v/v acetonitrile, 0.1% v/v formic acid) and then separated on an Easy Spray column (50 cm long, 75 μm inner diameter, 2 μm particles; Thermo Fisher Scientific, United States). Peptides were eluted using a gradient of 2–30% buffer B (80% v/v acetonitrile, 0.1% v/v formic acid) over 100 min, 30–40% buffer B for 20 min, 40–100% buffer B for 5 min and a wash at 100% B for 10 min at a constant flow rate of 250 nl/min. Total LC-MS/MS run-time was about 175 min, including the loading, linear gradient, column wash, and the equilibration.

LC-MS/MS data was acquired using the settings described below. The most abundant precursor ions from each survey scan that could be fragmented in 1 second (s) were dynamically chosen, where each ion was isolated in the quadrupole (0.7 *m/z* isolation width) and fragmented by higher-energy collisional dissociation (27% normalized collision energy). The survey scans were acquired in the Orbitrap at mass over charge ratios (*m/z*) of 375–1500 with a target resolution of 240,000 at *m/z* 200, and the subsequent fragment ion scans were acquired in the ion trap at a rapid scan rate. The lower threshold for selecting a precursor ion for fragmentation was 1.5 × 10^4^. Dynamic exclusion was enabled using a *m/z* resistance of 10 parts per million (ppm), a repeat count of 1, and an exclusion duration of 15 s.

#### Data Processing

All spectra were processed using MaxQuant (v1.6.7, Max Planck Institute) using the imbedded Andromeda search engine. Searches were performed against a subset of the SwissProt database set to *E. coli* K-12 (4519 sequences). The following search parameters were used: Carbamidomethyl (C) was selected as a fixed modification, Oxidation (M) and Acetyl (Protein N-term) as variable modifications, fragment ion mass resistance of 0.5 Da, parent ion resistance of 20 ppm, and trypsin enzyme with up to two missed cleavage. False discovery rates were set up using 0.01 for peptides, 0.01 for proteins, and at least 1 razor peptide per protein. Label free quantification (LFQ) was enabled for Quantitation. Resulting LFQ intensities were imported into Perseus v1.6.5 (Max Planck Institute) (Tyanova et al., 2016). In Perseus the data was Log2 transformed. All proteins that did not have a least three valid log2 LFQ intensities from ID were filtered out. Proteins present were assessed for significance using volcano plots with a modified Student’s *t*-test (false discovery rate of 0.05; S_0_ = 0.1). Enrichment analysis of protein-to-protein interactions was performed with Cytoscape v3.7.2 (Shannon et al., 2003) using the StringApp v1.5.0 software package (Doncheva et al., 2019). To analyze functional pathways involved in adaptation, significant proteins were categorized using the KEGG pathway database with ClueGO v2.5.5 (Bindea et al., 2009) application for Cytoscape. Default parameters for KEGG analysis were used, with network specificity set at “medium” and 50% overlap of genes for the group merge.

### RNA-Seq Transcriptomic Analysis

Transcriptomic analyses were preformed with 10 mL WT and CHXR1 cultures grown to mid-log phase (OD_600 *nm*_ = 0.5 units) in biological triplicate, where CHXR1 cultures were grown with CHX selection as described for proteomic analyses. Cells reaching mid-log were immediately stored on ice and total RNA was extracted using a bacterial RiboPure RNA purification isolation kit (Ambion Inc, TX, United States) according to the manufacturer’s protocol for Gram-negative bacterial extraction yielding 25–50 μg of RNA/sample. Ribosomal RNA was depleted from these samples using MICROB*Express* Bacterial mRNA Enrichment kit (Ambion Inc, TX, United States) based on the manufactures recommended standard protocol. mRNA sequencing analyses were performed by LC Sciences total RNA sequencing services (StateTexas, United States) using an Illumina NovaSeq 6000 for paired-end sequencing. RNA integrity, quality control and quantification analyses were performed using an Agilent Technologies 2100 Bioanalyzer with high sensitivity DNA chip.

Transcript sequences were bioinformatically analyzed by LC Sciences using the following workflow. Raw transcript sequence reads were assembled using Cutadapt (Martin, 2011) and in-house perl scripts to remove adaptor contaminated reads, low quality bases and undetermined bases. Sequences were verified using FastQC^5^ and bowtie2 (Langmead and Salzberg, 2012) was used to map reads to the *E. coli* BW25113 genome (GenBank accession number CP009273.1). Mapped reads for each bioreplicate were assembled using StringTie software (Pertea et al., 2015) and transcriptome datasets were merged to reconstruct a comprehensive transcriptome using perl scripts and gffcompare (Pertea and Pertea, 2020). StringTie (Pertea et al., 2015) and R statistics package edgeR^6^ were used to estimate the expression levels of all transcripts (Supplementary Table 6). The statistical significance (*p*-values < 0.05) of differentially expressed mRNAs based on their log2(fold change; FC) > 1 or log2(FC) < -1 were assessed using R statistics edgeR v3.14.0 package. Agglomerative hierarchical heatmaps of selected differentially transcribed genes listed in Supplementary Table 6 was generated using R statistics software v 4.0.3 (R Development Core Team, 2008) using the heatmap.plus (Day, 2012) package (Figure 5).

### Plasmid Complementation and Gene Deletion Assays of *E. coli* Strains and Isolates

Plasmid complementation assays were performed using chemical competent cell preparations of *E. coli* BW25113, Keio collection gene deletion mutants (listed in Table 1), and CHRX1-3 isolates using the RbCl_2_ protocol described in Green and Rogers (2013). ASKA collection strains AG1 (ME5305; pCA24N–), JW2343-AM (pMlaA–), JW5490-AM (pYghQ–), JW5248-AM (pMarR–), JW4276-AM (pFimE), JW3480-AM (pGadE), and JW5013-AM (pCdaR) were used to extract and purify their respective plasmid clones, using plasmid DNA extraction kits and isolation protocols from BioBasic Inc (ON, Canada). pCA23N(–) plasmids add an in-frame amino-terminal hexahistidine affinity tag to each cloned gene as described by Kitagawa et al. (2005). Plasmids were individually transformed into each *E. coli* strain listed above using the protocol described by Green and Rogers (2013) and transformants were selected and grown on LB medium with 30 μg/mL CM to maintain plasmid selection. Plasmids were re-isolated from each transformant to verify proper plasmid transformation. All transformants were examined using the same broth microdilution AST methods as described in sections above, at increasing CHX concentration ranges (0.5–16 μg/mL) to calculate any differences in MIC values between the transformants. All AST experiments were performed in triplicate for each transformed isolate or strain (Table 5).

## Data Availability Statement

The datasets presented in this study can be found in online repositories. The names of the repository/repositories and accession number(s) can be found in the article/Supplementary Material.

## Author Contributions

DCB and NC designed the study, where NC performed the adaptation experiment. NC, KG, and BG performed the AST and evaluated MIC data. KG and BG measured the growth curve data. KG measured the stability data. NC and SR performed the Genomic DNA extractions and WGS SNV identification. SR generated and analyzed the WGS phylogenetic trees. BG and SR performed the SEM imaging with DRB, SH, and TB. BG and SR prepared all proteomic samples for nano LC-MS/MS collection by PC and GW. KG, BG, and SR extracted the transcriptome RNA. DCB conducted sequencing by LC Sciences services and analyzed the data. BG and SR performed CHXR and WT plasmid transformant and gene deletion MIC testing. BG performed impermeant fluorescent dye CHXR and WT cell experiments. BG, SR, and DCB analyzed the all data and prepared manuscript figures. DCB wrote the manuscript draft in consultation with KG, BG, and SR. DCB and GZ edited the manuscript. All the authors read and approved the final manuscript.

## Conflict of Interest

The authors declare that the research was conducted in the absence of any commercial or financial relationships that could be construed as a potential conflict of interest.

## Footnotes

1 https://clsi.org/

2 https://www.eucast.org/

3 cgsc2.biology.yale.edu

4 https://microbesng.com/

5 http://www.bioinformatics.babraham.ac.uk/projects/fastqc/

6 https://www.rdocumentation.org/packages/edgeR
