# Supplementary material for: Phenotypic and Multi-Omics Characterization of Escherichia coli K-12 Adapted to Chlorhexidine Identifies the Role of MlaA and Other Cell Envelope Alterations Regulated by Stress Inducible Pathways in CHX Resistance
Source: Front Mol Biosci. 2021 May 19;8:659058. doi: 10.3389/fmolb.2021.659058 (PMC8170033; doi:10.3389/fmolb.2021.659058)
Supplement: Supplementary file 2 [file Data_Sheet_2.pdf]

Supplementary Figure Files

Title: Phenotypic and multi-omics characterization of *Escherichia coli* K-12 adapted to chlorhexidine identifies the role of MlaA and other cell envelope alterations regulated by stress inducible pathways in CHX resistance

Authors: Branden S.J. Gregorchuk[a], Shelby L. Reimer[a], Kari A.C. Green[a], Nicola H. Cartwright[a], Daniel R. Beniac[b], Shannon L. Hiebert[b], Timothy F. Booth[b], Patrick M. Chong[b], Garrett R. Westmacott[b], George G. Zhanel[a], Denice C. Bay[a#]

Institutional Addresses:

a. Department of Medical Microbiology and Infectious Diseases, University of Manitoba, Winnipeg, Manitoba, Canada
b. National Microbiology Laboratory, Public Health Agency of Canada, Winnipeg, Manitoba, Canada

Running Title: *mlaA* participates in *E. coli* CHX resistance

[#]Corrisponding author, Denice C. Bay, denice.bay@umanitoba.ca
Assistant Professor
Rm 514C Basic Medical Sciences Bldg.
Department of Medical Microbiology and Infectious Diseases
University of Manitoba
745 Bannatyne Avenue
Winnipeg, MB, Canada R3E 0J9
Tel: (204) 977-5679
Fax: (204) 789-3926

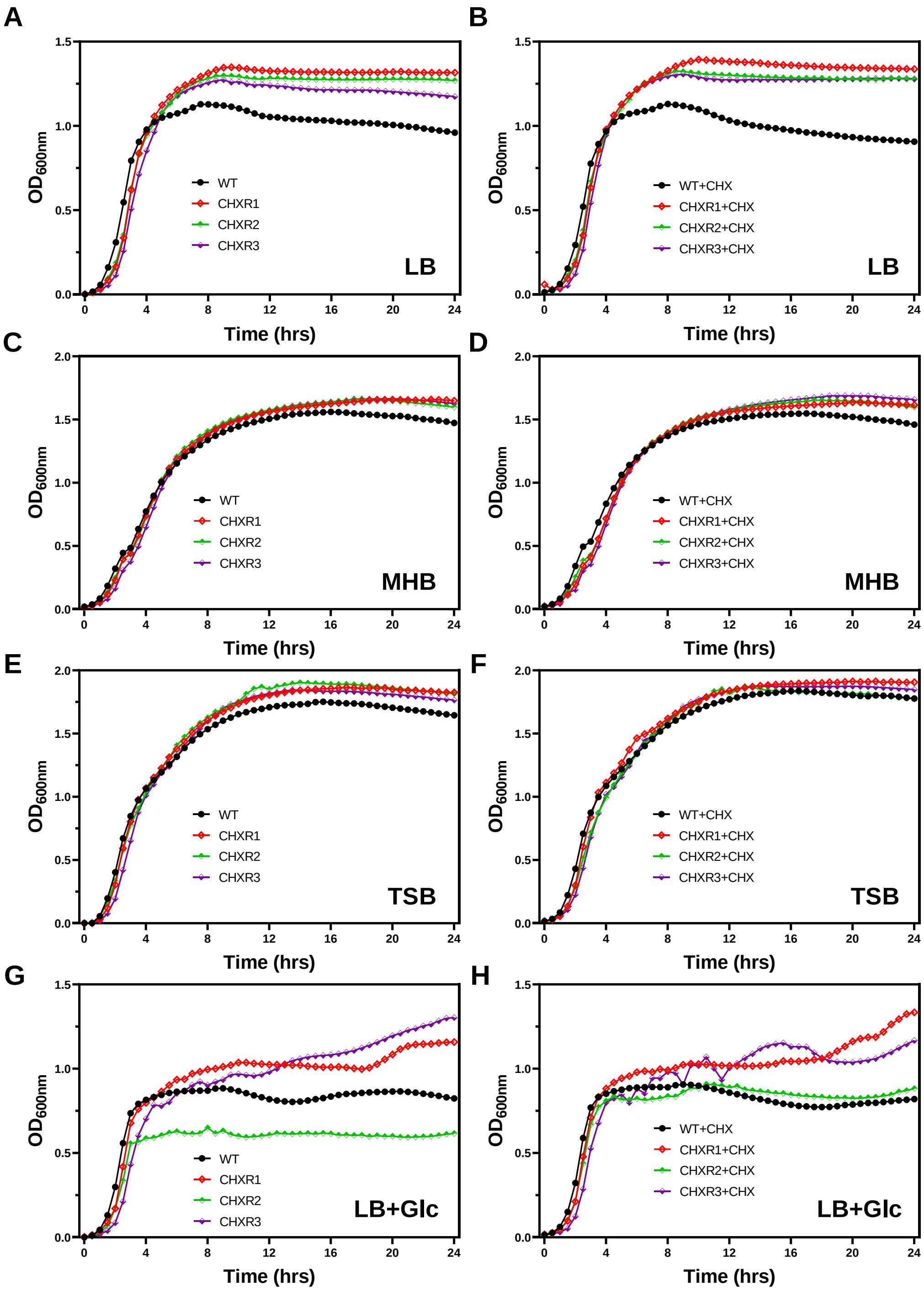

Figure S1. Optical density (OD_{600nm}) measured growth curves of BW25113 (WT) and individual CHXR1-3 isolates grown in rich media with added 0.4 µg/mL CHX (B, D, F, H) and without (A, C, E, G) CHX. Each growth curve shows the mean of two technical duplicate growth curves of each CHXR isolate and WT. The error bars from each growth curve were omitted for clarity. The growth media used to grow each isolate/strain is listed in the bottom right-hand corner of each panel where each medium is defined is as follows: Luria-Bertani (LB), LB + 0.4% w/v glucose (LB+Glc), Mueller Hinton broth (MHB) and tryptic soy broth (TSB).

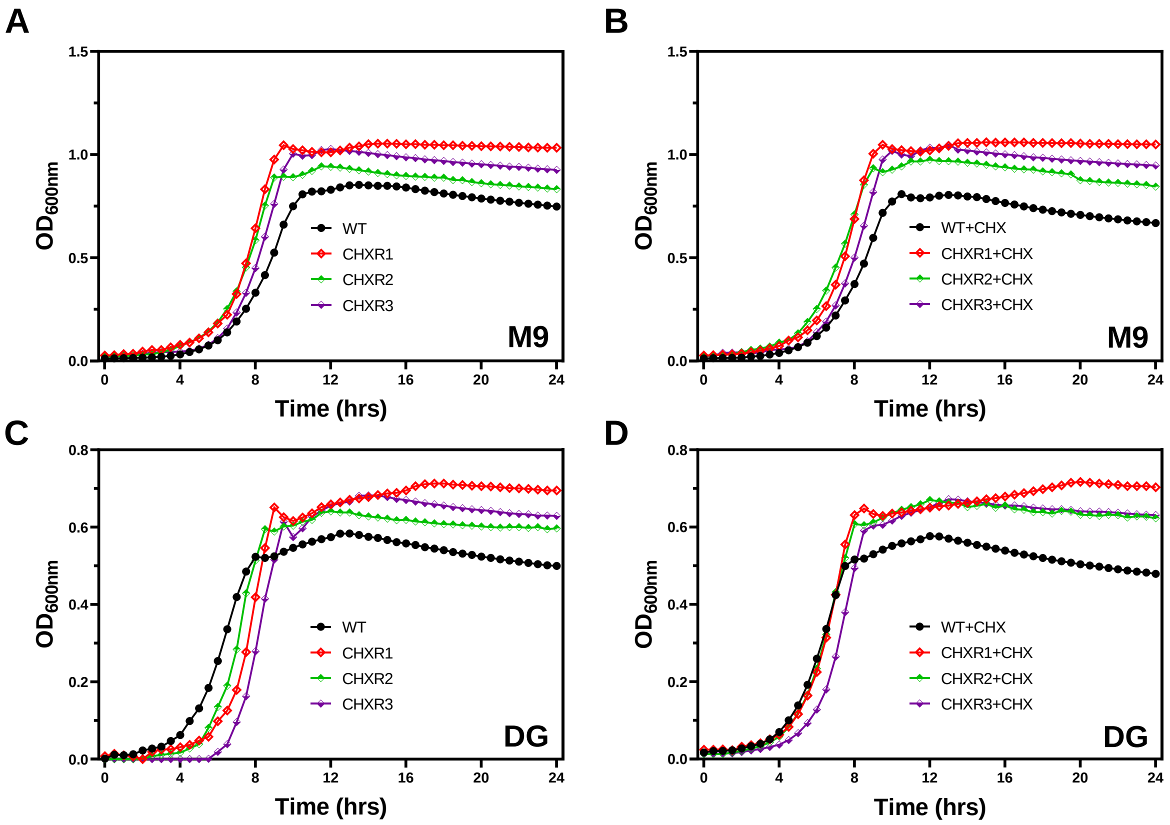

Figure S2. Optical density (OD_{600nm}) measured growth curves of BW25113 (WT) and individual CHXR1-3 isolates grown in minimal media with added 0.4 µg/mL CHX (B, D) and without (A, C) CHX. Each growth curve shows the mean of two technical duplicate growth curves of each CHXR isolate and WT. The error bars from each growth curve were omitted for clarity. The growth media used to grow each isolate/strain is listed in the bottom right-hand corner of each panel and media is defined is as follows: Davis-Glucose (DG) and minimal 9 salts (M9) media.

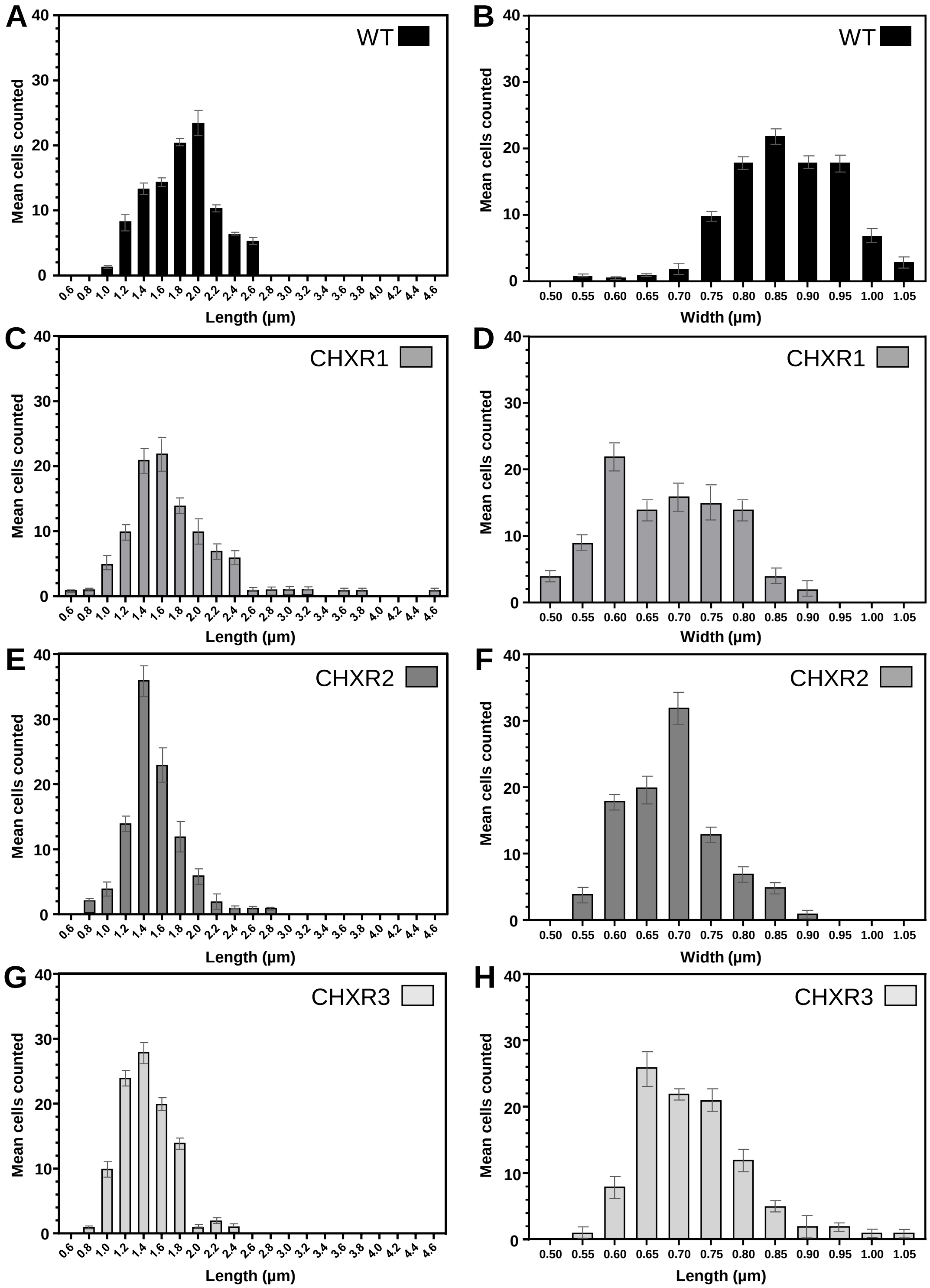

Figure S3. Histograms of *E. coli* WT and CHXR isolate mean cell lengths and width distributions as determined from SEM measurements of 100 cells using ImageJ. Each panel represents the mean number of cells measured from SEM images of 2 biological replicates (error bars). The isolate or strain measured is indicated in the right-hand panel. Bars represent the binned lengths or widths of 100 cell measurements as indicated.

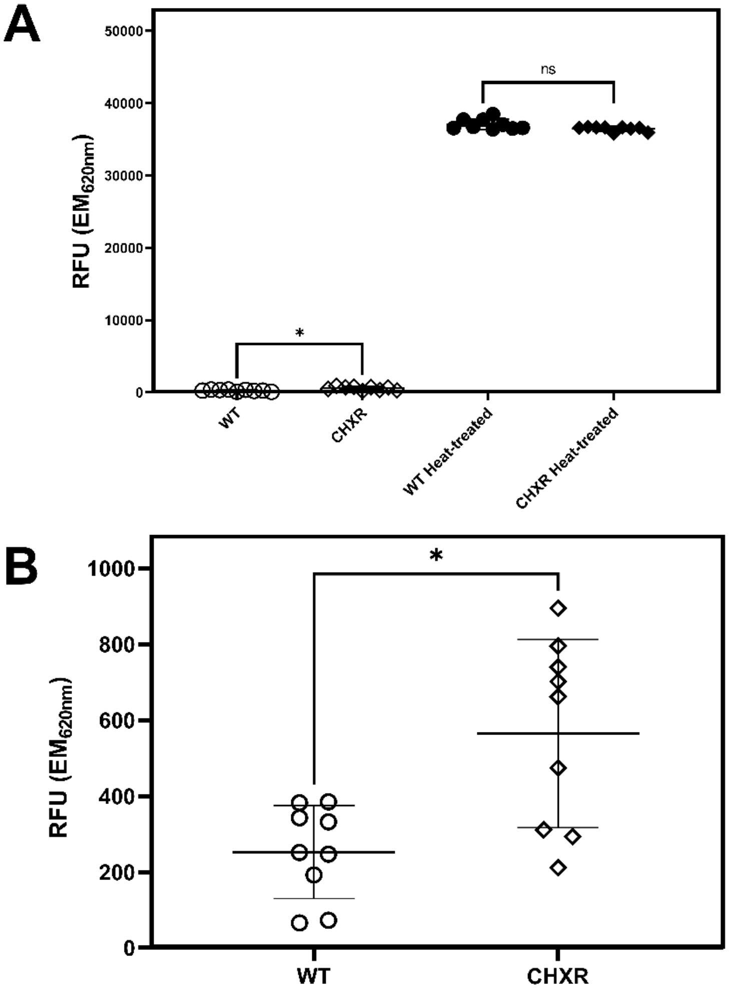

Figure S4. Propidium iodide dye fluorescent RFU emission values determined from mid-log cell preparations of CHXR1 isolates and WT after 30-min incubation. In both panels A-B, CHXR1 isolates (biological replicates) and WT baseline subtracted RFU values are shown individually (n=9), where each culture replicate was diluted to final OD_{600nm} of 0.2 units in phosphate buffered saline with 2.0 µg/ml propidium iodide (final concentration). **A)** RFU of CHXR1 (CHXR) and WT cell preparation of live and heat-inactivated samples. Live and heat-treated samples compare the maximum dye penetration rate of heat-permeabilized/ dead cell preparations as a positive control. **B)** Comparison of RFU values of live WT and CHXR. RFU and standard deviations bars within the samples are displayed. Mann-Whitney tests were performed comparing WT to CHXR1 as well as heat-treated WT and heat-treated CHXR1 with P<0.05 being significant and indicated with an asterisk.

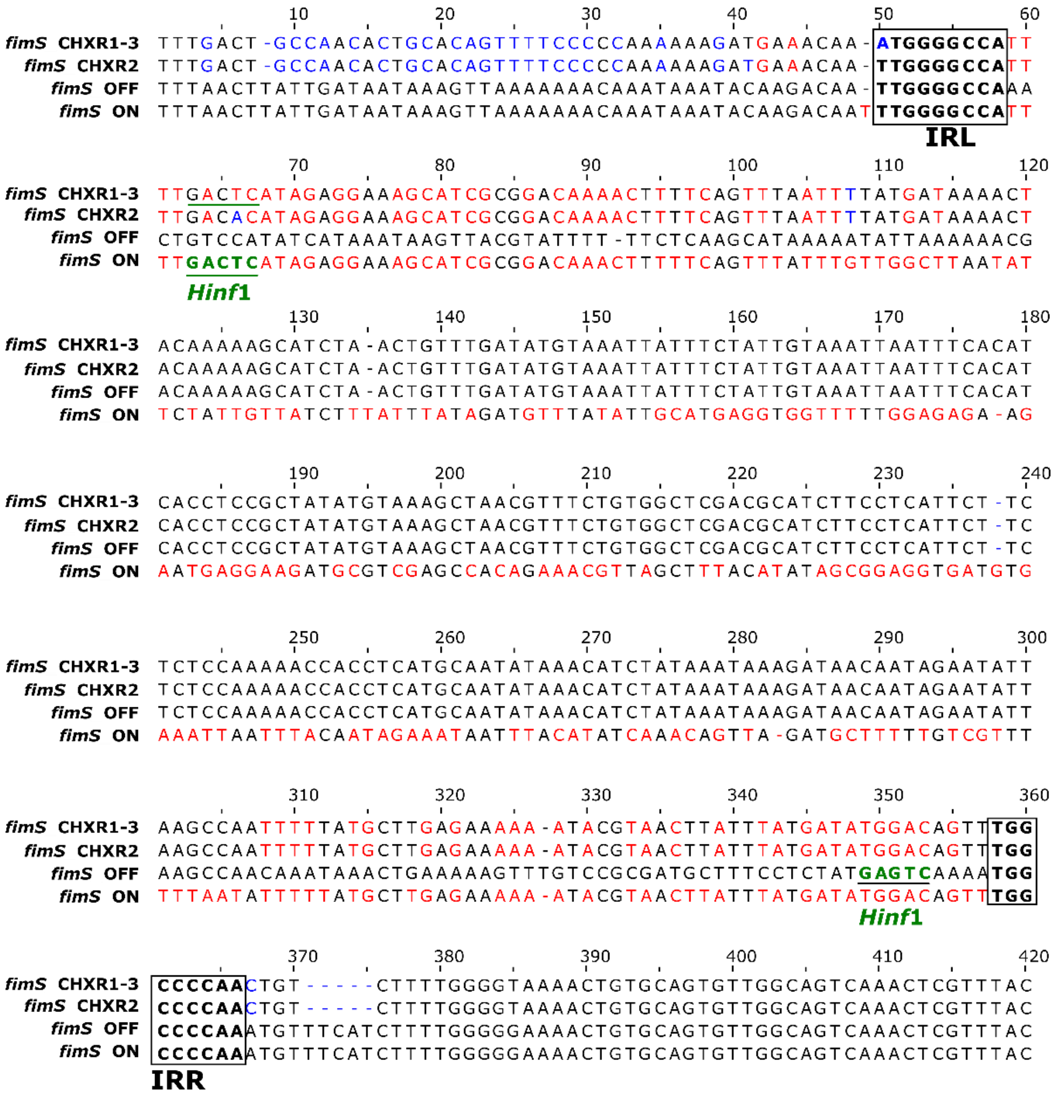

Figure S5. Multiple nucelotide sequence alignment of the hybrid *fimS* promoter switch region of *E. coli* WT and CHXR which has both ON and OFF *fimS* switch homology. Nucleotide (nt) regions corresponding to *fimS* promoter ON and OFF configurations (due to FimE recombinase activity) are shown. The *fimS* promoter is shown here is located in the intergenic region between the *fimE* stop codon until the *fimA* start codon (CP_009273.1; 4532450-4532932 nt). ON and OFF sequences are based previously published *fimS* regions (Shembri *et al*. 1998). CHXR1 (NZ_CP069132.1) and CHXR3 (NZ_CP069134.1) genomes were identical in sequence and are shown as "*fimS* CHXR1-3" in the alignment; the *fimS* region of CHXR2 genome (NZ_CP069133.1) was distinct from the *fimS* sequence region and is shown separately as CHXR2. Boxed nt regions show the inverted right repeat (IRR) and IRL inverted left repeat sequences that are switched in *fimS* by FimE. Red nt show sequence identity to the *fimS* ON switch configuration whereas blue nt indicate unique nt changes in CHXR1/3 or CHXR2 sequences only. Black font indicates sequence identity to the *fimS* OFF switch configuration. Green underlined nt indicate *Hinf*1 restriction sites distinguishing the ON and OFF switches. Notably, CHXR1 and CHXR3 only had one *Hinf*1 restriction site at the same aligned position as expected for the *fimS* ON switch configuration and CHXR2 had no *Hinf*1 site.

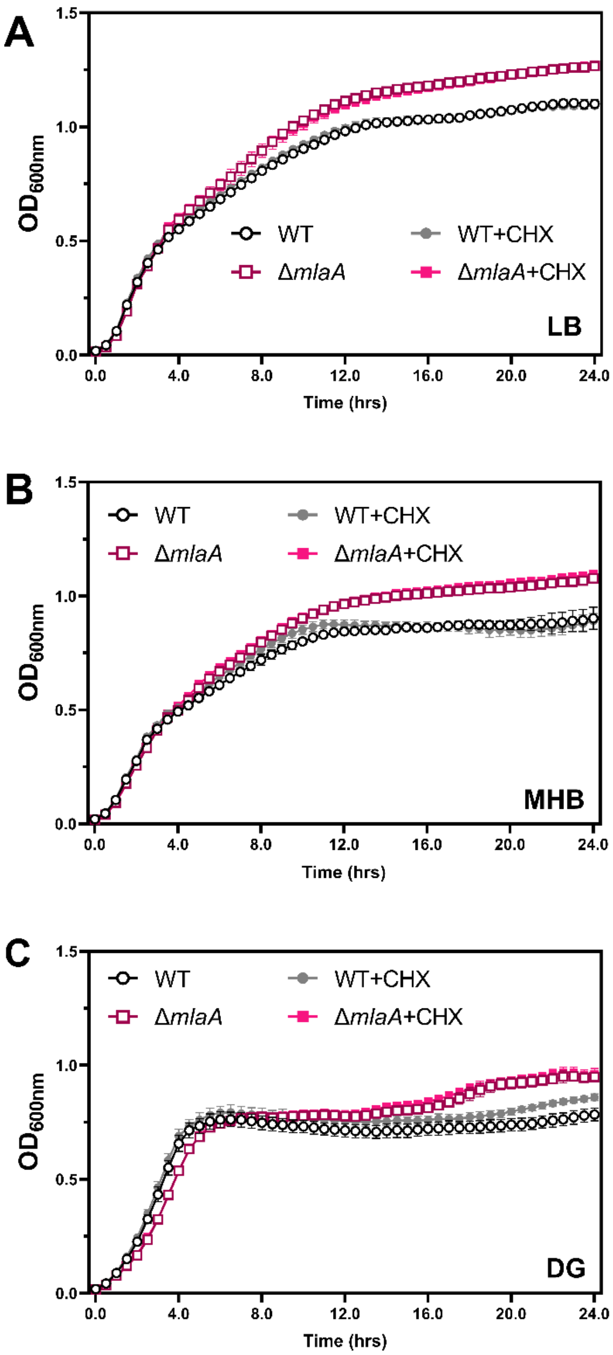

Figure S6. A summary of optical density (OD_{600nm}) 24 hr growth curves of *E. coli* WT BW25113 (WT) and JW2343-KC (Δ*mlaA*) at 37ºC. Panels A-C show LB, MHB and DG growth curves respectively of WT and Δ*mlaA* strains grown with and without 0.4 µg/mL CHX. WT without CHX (black circles), WT with 0.4 µg/mL CHX (grey filled circles), Δ*mlaA* without CHX (pink squares), and Δ*mlaA* with CHX (pink filled squares) are shown in all panels. Error bars represent the standard deviation of 4 biological replicates. Statistical analysis of WT and Δ*mlaA* using Kruskal-Wallis tests did not show any significant differences in OD_{600nm} values in rich or minimal media assessed ($P>0.05$).
